# CircMVP promotes METTL3 activation mediated CTNNB1 m6A modification in the inhibition of colorectal cancer in B7-H3 dependence antitumor immunity

**DOI:** 10.7150/ijbs.105324

**Published:** 2025-01-01

**Authors:** Fang Wang, Qian Wang, Yongfeng Wu, Zebo Huang, Xincao Zhong, Hunan Wang, Chen Yang, Yan Qin, Xiaowei Qi, Xiaosong Ge, Yong Mao

**Affiliations:** 1 Department of Cancer Diagnosis and Treatment Center, Affiliated Hospital of Jiangnan University, Wuxi 214122, China.; 2 Laboratory of Oncology Precision Diagnosis and Treatment, Wuxi Medical College of Jiangnan University,Wuxi 214122, China.; 3 Key Laboratory of Integrated Oncology and Intelligent Medicine of Zhejiang Province, Affiliated Hangzhou First People's Hospital, Westlake University School of Medicine, Hangzhou 310003, China.; 4 Department of Plastic Surgery, Sir Run Run Shaw Hospital, Zhejiang University School of Medicine, Hangzhou 310016, China.; 5 Department of Pathology department, Affiliated Hospital of Jiangnan University, Wuxi 214122, China.; Fang Wang, Qian Wang and Yongfeng Wu contributed equally.

**Keywords:** Colorectal cancer, circRNA, METTL3, B7-H3, Tumor immune evasion

## Abstract

The effect of immunotherapy for colorectal cancer (CRC) is limited due to anti-tumor immunosuppression. Circular RNAs (circRNAs) are also associated with tumor immunity. The aim of this study was to clarify the regulatory relationship between circRNA and anti-tumor immunosuppression in CRC. CircRNAs associated with CRC were identified using bioinformatic analysis and subsequently confirmed in clinical samples using qRT-PCR and *in situ* hybridization. The expression, clinical relevance, functional significance and clinical properties of circMVP in CRC specimens and cells were evaluated *in vitro* and *in vivo*. RNA pull-down, single-cell RNA sequencing, EMSA, RNA immunoprecipitation, chromatin immunoprecipitation, and polysome profiling assay were performed to confirm the underlying mechanism of circRNA. CircMVP (hsa_circ_0000688) expression was increased in CRC and correlated with poor prognosis in CRC patients. Increased circMVP expression activates proliferation, invasion, and tumorigenesis of CRC. In addition, we found that circMVP, by interacting with METTL3, stabilizes its expression in the nucleus and significantly enhances its mediated N6-methyladenosine (m6A) modification. Specifically, circMVP/METTL3 promoted the expression of β-catenin by directly acting on CTNNB1 mRNA. CircMVP/METTL3 further enhanced the expression of B7-H3 through the β-catenin signaling pathway. Notably, inhibition of circMVP expression significantly improved the efficacy of anti-B7-H3 immunotherapy in *in vivo* and *in vitro* models. CircMVP mediated CTNNB1 m6A modification by promoting METTL3 activation and inhibited B7-H3-dependent anti-tumor immune response in CRC. In conclusion, circMVP may be a predictor of CRC immune evasion and a potential therapeutic target.

## Background

Colorectal cancer (CRC) is the prevailing malignancy globally and is the foremost cause of cancer-related mortality^1^. Radical surgery is the most common treatment for CRC^2^. Most patients with advanced CRC have a high metastasis rate at diagnosis and relapse after treatment, and traditional treatment is not effective and the prognosis is poor^3^,^4^. Recently, immune checkpoint blockade (ICB) treatments targeting the inhibition of Programmed cell death protein 1 (PD-1) and Programmed cell death 1 ligand 1 (PD-L1) have exhibited remarkable efficacy in the management of solid tumors^5^. However, the response of CRC to PD-1/PDL-1 immunotherapy is limited, suggesting the need to explore immune checkpoint targets 6,7. Understanding the molecular mechanisms underlying ICB regulation in CRC can augment the clinical effectiveness of ICB therapy.

Circular RNAs (circRNAs) are abundant, conserved, ubiquitous, and purposeful noncoding RNAs characterized by a covalent closed-loop structure composed of single-stranded RNAs 8,9. They are produced through back-splicing of their host genes and exhibit exceptional stability owing to the absence of 5′ caps and 3′ tails. Based on their localization and interactions with other molecules, circRNAs regulate transcription and splicing, influence mRNA stability and translation, influence protein function and metabolism, and even serve as a template to encode peptide segments in a variety of biological and pathophysiological environments 10. CircRNAs are involved in the regulation of tumor cells and the tumor microenvironment (TME), thereby affecting anti-tumor immunity^11^,^12^. Nevertheless, substantial research remains indispensable prior to the implementation of circRNAs in clinical settings.

Recently, several studies have reported that circRNAs play a key role in regulating the tumor immune microenvironment 13,14. Dysregulation of circRNAs is commonly observed in malignancies, raising the hypothesis that their imbalanced expression significantly contributes to the initiation and advancement of tumorigenesis 15. Nevertheless, there is a paucity of information pertaining to the participation of circular RNAs in immunosuppressive signaling, specifically with regard to the modulation of immune checkpoint expression and the prospective efficacy of immune checkpoint blockade.

This study demonstrated that a specific circRNA, circMVP (hsa_circ_0000688), is upregulated in CRC, and its overexpression facilitates cancer progression and immunosuppression. Mechanistically, circMVP combined with METTL3 regulates m6A methylation of CTNNB1 mRNA, upregulates β-catenin protein expression, and activates B7-H3 expression. Inhibition of circMVP expression and blockage of the B7-H3 checkpoint effectively inhibited CRC development and improved the efficacy of immunotherapy.

## Materials and methods

### Clinical samples

Parafin sections extracted from the tumors and neighboring healthy tissue samples of 162 CRC patients (surgeries conducted between 2012 and 2017) were acquired after obtaining signed informed consent. The protocol, duly approved by the Department of Surgery and Oncology at the Affiliated Hospital of Jiangnan University, strictly adhered to all pertinent ethical regulations. Detailed data pertaining to patient gender and clinicopathological parameters are shown in Table S1. This part of the study was approved by the Ethics Committee of Affiliated Hospital of Jiangnan University (no.LS2020058).

### Mouse tumor model

Five-week-old BALB/c nude mice were purchased from the Shanghai SLAC Laboratory Animal Center (Shanghai, China) and treated with a subcutaneous injection of 1.0 × 10^6^ DLD1 or HCT8 cells. For the *in vivo* C57BL/6 mice tumorigenesis experiments (n = 5 per group), mice were treated with a subcutaneous injection of 1.0 × 10^6^ MC38 cells (n = 5 per group). With regard to ICB and combination therapy treatments, tumor-bearing mice were treated with an intraperitoneal injection of 200 μg of the anti-mouse B7-H3 antibody Omburtamab (1895083-75-6, Selleck, China) or mouse IgG1 isotype control (MOPC-21, Selleck, China) on the indicated days. A cross-sectional study was performed to evaluate tumorigenesis and detect circMVP, METTL3, and B7-H3 expression 4 weeks after tumor cell injection (5 per group were used). The remaining mice (5 per group) were maintained until mortality or until the tumor reached a maximum subcutaneous size of 2000 mm^3^.

All mice were housed in the Jiangnan University Laboratory Animal Center under specific pathogen-free (SPF) conditions. They were raised and nurtured within the same establishment, occupying the same unit in an animal housing chamber equipped with regulated temperature (22°C), photoperiod (12-hour light/12-hour dark cycle), and unrestricted availability of standard mouse chow and water, excluding those utilized in select experiments described in this manuscript. The ethical utilization of all mice was conducted in strict adherence to the guidelines set forth by the Jiangnan University Animal Ethics Committee (JN. No. 20231030m0960420).

### Cell lines

HCT8 cells (ATCC, CCL-244), HCT116 (ATCC, CCL-221), RKO (ATCC, CCL-2577), MC38 (EK-bioscience, CC-Y2123), 4T1 (ATCC, CRL-2539), 293T (ATCC, CRL-11268), B16 and CT26 cells (provided by Professor Yushi Yao, Zhejiang University, Hangzhou, China) were nurtured using Dulbecco's modified Eagle's medium (DMEM), DLD1 (ATCC, CCL-247) and iBMDM cells (provided by Professor Dajing Xia, Zhejiang University, Hangzhou, China) were cultured in RPMI 1640 medium. All cells were cultured with 10% fetal bovine serum (FBS; Clark, FB15015) and 100 U/ml penicillin-100 μg/ml streptomycin. The last STR test of the main cell lines involved in this design was commissioned by BIOWING (Shanghai, China) in January 2022.

### CCK-8, colony formation and transwell assays

DLD1 and HCT8 cells were carefully selected to facilitate the knockdown of circMVP. Cells were carefully placed in 96-well plates and cultivated for 24, 48, 72, and 96 hours. At specific intervals, a volume of 10 µL CCK-8 reagent (Vazyme) was meticulously introduced into each well of a 96-well plate, followed by an incubation period of 2 h. Absorbance was measured at a wavelength of 450 nm using a microplate reader.

To ensure optimal conditions, 200 cells were carefully seeded into each well of 6 well plates and cultivated for a meticulously calculated span of 10 to 15 days. Following this, the cells were diligently fixed with 4% paraformaldehyde and thoroughly stained with crystal violet solution to evaluate their propensity for colony formation.

For the assessment of cell migration and invasion, Transwell plates with a pore size of 8 μm were delicately employed, employing the instructions provided by the manufacturer (12.5% Matrigel was used for invasion assays). In the upper chamber, the cells were meticulously plated in 300 μL of serum-free medium and then inserted into a 24-well plate at a concentration of 5×10^4^ cells per well. The lower chamber was meticulously filled with 700 μL of medium containing 10% fetal bovine serum. After a meticulous incubation period of 24 h, non-migrated cells were gently removed from the chamber using a cotton swab and subsequently stained with 2% crystal violet. Finally, the migrated and invaded cells were visually captured and recorded using the awe-inspiring capabilities of an Eclipse Ci-L Microscope (Nikon).

### Western blot

Protein extracts were acquired from cells implicated in this inquiry by employing the RIPA lysate kit (P0013B, Beyotime). Quantification was performed using a BCA Protein Assay Kit (P0012, Beyotime), followed by subjecting them to electrophoresis on an SDS-PAGE gel. The protein samples were then transferred onto a nitrocellulose filter membrane (Cytiva) in equal proportions, blocked with 5% skim milk (Sangon) in TBST, and incubated with primary antibodies at 4 °C overnight. The membrane was then exposed to HRP-conjugated secondary antibodies, developed using chemiluminescence reagents, and visualized using a molecular imaging system (Tanon).

### Northern blot

CircMVP-specific probes (Supplementary Table S2) were synthesized by Fenghbio (China). The circRNA was purified and enriched by RNase R treatment, followed by polyadenylation and depletion of poly(A)+ RNA. Electrophoresis was performed in a 2% agarose gel infused with 4% formaldehyde and subsequently translocated onto an excited nitrocellulose membrane (Cytiva). Following UV cross-linking, it was harmonized with FITC-labeled probes and underwent radioactivation on X-ray film. CircMVP was assessed using a GeneRuler DNA ladder (SM0332, ThermoFisher) as a benchmark, which was Cy3-coupled according to a tailor-made procedure (Telenbiotech).

### RNase R treatment

Total RNA (2 μg) was incubated for 15 min at 37 °C with 5 U/μg RNase R (Epicenter Technologies, Madison, WI) and then analyzed by RT-PCR^16^.

### Flow cytometry

To prepare a single-cell suspension, the collected tumors were digested using collagenase IV at a concentration of 1 mg/ml, hyaluronidase at a concentration of 0.2 mg/mL, and DNase I at a concentration of 0.15 mg/ml. This digestion process took place at a temperature of 37°C on a rotating platform for a duration of 1 h, after which the mixture was filtered through a cell strainer with a pore size of 70 mm. The cells were then washed with cold PBS. Following this, the red blood cells were lysed using the MultiScience product, whereas the dead cells were labeled using the Invitrogen Fixable Viability Dye Kit. The cells were washed again with a staining buffer composed of PBS supplemented with 2% FBS and 1 mM EDTA. The cell suspension was then resuspended in the same staining buffer, with the addition of CD16/CD32 block antibody from MultiScience, and incubated for 20 min. Subsequently, specific antibodies targeting membrane molecules at appropriate dilutions were added to the suspension and incubated for 30 min on ice in the absence of light. Finally, intracellular staining was conducted using a FoxP3/Transcription Factor Staining Buffer Kit from MultiScience.

Distinct immune cell populations were identified and gated in this study: EpCAM+ cell populations (Invitrogen), CD45+ cell populations, CD4+ T cells (CD3+ CD4+), CD8+ T cells (CD3+ CD8+), NK (Natural Kill) cells (CD3- NK1.1+), tumor-associated macrophages (TAMs) (CD11b+ F4/80+), and M2 TAMs (CD11b+ F4/80+ CD206+). Prior to analysis, the cells were fixed using a Fixation/Permeabilization Kit from MultiScience for 30 min at room temperature. Subsequently, the cells were washed twice with diluted permeabilization buffer from MultiScience. The addition of antibodies then took place, followed by overnight incubation at 4°C. To ascertain precise compensation adjustments, we used either single-staining specimens or UltraComp eBeads Compensation beads from Invitrogen. Flow cytometry data were acquired using Beckman Coulter DxFlex or the multicolor flow cytometer BD Fortessa, and subsequent analysis was performed using FlowJo software (V10).

### RNA extraction, reverse-transcription, and quantitative PCR

RNA extraction was performed using Trizol reagent (B511311, Sangon), followed by reverse transcription of 1000 ng of total RNA using the High Capacity cDNA Reverse Transcription Kit (TaKaRa, Japan) according to the manufacturer's instructions. qRT-PCR analysis was conducted using a Bio-rad CFX-96 Quantitative PCR System (Applied Biosystems, Foster City, CA, USA). The expression of β-actin was used as a reference to normalize the initial mRNA concentration in tissues and cells. The target gene expression was calculated using the 2^-ΔΔCT^ method. The primer sequences are listed in Supplementary Table S2.

### ISH and FISH

RNA fluorescence *in situ* hybridization (FISH) was conducted using a FISH Kit (C10910, RiboBio) following the manufacturer's instructions. The RNA probe was designed using PaintSHOP (https://oligo.shinyapps.io/paintshop/) to specifically target the encompassed region. FITC-labeled and biotin-labeled probes designed to bind to circMVP were synthesized by RiboBio Technology. The mesmerizing fluorescence emitted from the samples was captured using an Olympus IX73 Microscope. *In situ* hybridization (ISH) was performed on paraffin-embedded sections of CRC tissues or animal tumors, adhering to a previously described protocol^17^. Briefly, the slices were digested using pepsin and then hybridized with digoxin labeled nucleotide sequences (Synbio-Tech, China) overnight at 37°C. The sections were then stained with DAB (Diaminobenzidine) staining the polymer-horseradish peroxidase (HRP) complex by hybridization, and hematoxylin for nuclear staining. Finally, remnant RNA expression was captured and immortalized through the lens of a distinguished IX73 Microscope (Olympus).

### Immunohistochemistry (IHC) and immunofluorescence (IF) staining

Paraffin-embedded CRC samples were sliced into slides with a thickness of 0.5 μm. To retrieve antigens, a pressure cooker was used to immerse the samples in a 0.01 M citrate buffer (pH 6.0) for 20 min. For IF assay, the cells were fixed at room temperature for 20 min using a 4% solution of paraformaldehyde and permeabilized on ice for 5 min with 0.05% Triton X-100 in PBS. The samples were then blocked with a 2% BSA solution in PBS at room temperature for 1 h and incubated overnight at 4 °C with METTL3, β-catenin, YTHDF1, and B7-H3. Subsequently, fluorescent dye labeled antibodies or secondary antibodies conjugated with HRP were administered to the samples for 1 h at ambient temperature. For multiplex IF staining was performed using a multiplex fluorescence IHC staining (abs50013, ABSIN). Nuclei were counterstained with DAPI and images were captured using either laser scanning confocal microscopy (FV1200, Olympus) or fluorescence microscopy (IX73, Olympus).

According to previous studies, the scoring of METTL3, β-catenin, YTHDF1, and B7-H3 staining was based on the coverage of tumor areas and the percentage of positive staining for the detected proteins^18^. Immunodetection was achieved using 3,3N-diaminobenzidine tetrahydrochloride (DAB), and the nuclei were counterstained with hematoxylin. The staining scores for METTL3, β-catenin, and B7-H3 were determined based on the intensity and proportion of positive cells in 3 randomly selected fields under ×40 objective. The proportion of positively stained tumor cells in the sections was graded as follows: 0 (no positive cells), 1 (< 25%), 2 (26-50%), 3 (51-75%), and 4 (> 76%). The staining intensity was recorded on a scale of 0 (no staining), 1 (light staining), 2 (moderate staining), and 3 (dark staining). The staining index (SI) was calculated using the following formula: SI = staining intensity × proportion of positively stained cells. The METTL3 score was determined based on the percentage of positive staining in the nucleus within the tumor area^19^. The protein expression signals were excited and recorded using Digital Slide Scanner (OptraScan). Please refer to Supplementary Table S3 for the list of antibodies used.

### Plasmid, lentivirus construction, RNA interference and transfection

Human and murine circMVP (hsa_circ_0000688) and sh-circMVP plasmids were selected and inserted into the pLV-ciR-Puro vector to ensure stable overexpression and knockdown, respectively, while employing an empty plasmid as a control. Circular RNA was synthesized and utilized *in vitro* using an elegant Circular Synthesis Kit (R0722, Beyotime). si-circMVP and si-METTL3 were purchased from GeneChem (Shanghai, China). Transfection of siRNAs and plasmids was performed using the Lipofectamine 3000 kit (Invitrogen) according to the manufacturer's instructions. All sequences are listed in Supplementary Table S2.

### Extraction of cytoplasmic and nuclear proteins

Extraction of cytoplasmic and nuclear proteins was performed using PARIS^TM^ Nuclear and Cytoplasmic Extraction Reagents (AM1921, Thermo Scientific), according to the manufacturer's instructions.

### RNA pull-down

*In vitro* transcription and biotin-labeled circMVP, circMVP-antisense, and CTNNB1 mRNA were conducted using a PCR instrument (Bio-Rad) with an SP6/T7 Biotin Labeling Kit (R7061, Beyotime). Subsequently, the resulting biotin-labeled RNA was incubated with cell lysates overnight at 4°C. Subsequently, a combination of 30 µL streptavidin magnetic beads (Thermo Fisher) was introduced into the mixture, which was then incubated for 2 h at room temperature. Afterwards, a total volume of 200 μL wash buffer (D3308, Beyotime) was used to cleanse the magnetic beads at room temperature for 2 h, with the aim of acquiring a protein suspension. Subsequently, an addition of 50 μL 5×SDS loading buffer was implemented, and the mixture was incubated at 100°C for 10 min. The proteins were subsequently subjected to SDS-PAGE analysis and Coomassie blue staining. The specific bands of protein expression were sliced and the protein Mass Spectrum (MS) assays were detected to detect the specific circMVP binding proteins.

### RNA decay assay

The fluorescence signal was detected by gel electrophoresis of the amplified product and the stability of circMVP was analyzed.

### RIP (RNA immunocoprecipitation)

The RIP assay was conducted following the instructions of the RIP Kit (Bes5101, Bersinbio). The cellular lysate was combined with A/G magnetic beads conjugated to the designated antibodies or IgG antibody (30000-0-AP, ProteinTech) and the mixture was incubated overnight at 4°C. RNA was purified using proteinase K, and RNA extraction was performed using the RNA isolator Total RNA Extraction Reagent. The immunoprecipitated methylated RNAs were purified using the RNA Cleaner beads (12602ES08, Yeasen) 20. Ultimately, qualitative analysis was performed using PCR and agarose gel electrophoresis, whereas quantitative analysis was performed using qRT-PCR.

### EMSA

Biotin-labeled circMVP RNA probes were obtained by *in vitro* transcription using the Biotin RNA Labeling Mix (11685597910, Roche). According to the manufacturer's instructions, a chemiluminescence RNA EMSA kit (GS606, Beyotime) displacement experiment was performed, and a molecular imaging system (Tanon) was used for visualization.

### RNA sequencing and circRNA analysis

Total RNA was extracted from three CRC tissues and three adjacent normal tissues, as well as HCT8 tumor samples with upregulated circMVP and a negative control (NC) (three replicates per group), using the RNeasy kit (Qiagen). Extracted RNA was dissolved in RNase-free water. Subsequently, Novogene Corporation sequenced the RNA samples. Briefly, RNA degradation and contamination were monitored using 1% agarose gel electrophoresis. The RNA purity was assessed using a NanoPhotometer Spectrophotometer (IMPLEN). RNA integrity and quantification were assessed using the RNA Nano 6000 Assay Kit on the Bioanalyzer 2100 system (Agilent Technologies). For RNA sample preparation, 1 μg of RNA per sample was used as input material. The cleaved RNA fragments obtained were transcribed in reverse, giving rise to the ultimate cDNA compendium, following the instructions provided in the mRNA-Seq sample preparation kit (Illumina, San Diego, USA). The average size of the inserted segments in the paired-end libraries was 300 bp with a variation of ± 50 bp. Finally, paired-end sequencing was performed using the Illumina sequencing platform. The RNA-seq data obtained in this study have been deposited in the SRA database under accession numbers PRJNA1073597 and PRJNA1073597.

To identify and quantify the circRNAs, each sample was sequenced on the Illumina HiSeq platform and mapped to the reference genome (GRCh38) using Bowtie2 (https://bowtie-bio.sourceforge.net/bowtie2/index.shtml). Qualified reads were then processed using the FIND-CIRC v1.2.0 pipeline with default parameters^21^. CircRNAs were considered present if they had at least two unique back-spliced reads. Data analysis was conducted using the R software (version 4.0.1) and the edgeR package (version 3.42.4). The threshold for significant differences was set as log2| fold-change | ≥ 1 and *P* < 0.05.

### Single-cell RNA (scRNA) sequencing and analysis

Freshly extracted surgical colorectal cancer (CRC) samples and mouse tumor tissue were used to create cell suspensions following the manufacturer's instructions using the 10x Chromium kit (10x Genomics, Pleasanton, CA). Subsequently, library construction was performed, followed by sequencing on a NovaSeq 6000 platform (Illumina, Inc., San Diego, CA) at Oebiotech (Shanghai, China). Raw sequencing reads were converted into fastq format and evaluated for quality using the FastQC software v0.11.9, accessible at https://www.bioinformatics.babraham.ac.uk/projects/fastqc/. Cell ranger software, with its standard pipelines, was employed for sequence processing, including alignment to the reference genome (Homo sapiens: GRch38 or Mus musculus: mm10) using default parameters (https://support.10xgenomics.com/single-cell-gene-expression/software/pipelines/7.1.3/).

The expression profiles of single cells were clustered into groups based on their principal components using 20 components. The unsupervised graph-based clustering algorithm Louvain (resolution: 1.0/1.5) was used for this purpose. Clustering, normalization, differential gene expression analysis, and visualization were performed using Seurat 4.3.0. The 'FindClusters' function within Seurat implemented the community identification method for cell clustering. The Seurat 'FindMarkers' function was used to identify specific marker genes for each cell cluster. The clusters were further assessed for known gene signatures, including epithelial cells (EPCAM, Grb2, KRT8, KRT14, and ERBB2), T cells (KLRC1, CCR7, FOXP3, CTLA4, CD8B, CXCR6, and CD3D), NK cells (NCAM1, KLRB1, NCR1, and GZMB), macrophages (CD68, F4/80, ITGAM, ITGAX, and CSF1R), and B cells (MZB1, IGHA1, CD19, and AICDA). Differentially expressed genes (DEGs) within a particular cluster were identified by comparing cells within the said cluster to all other cells, employing the Wilcoxon Rank-Sum test. For cell-cell communication analysis, CellPhoneDB73 (v2.1.7) was employed with default parameters, utilizing normalized expression values as input. The output from CellPhoneDB was visualized using kt plots (https://www.github.com/zktuong/ktplots/). The RNA-seq data presented in this study were deposited in the Sequence Read Archive (SRA).

### MeRIP sequencing analysis

Selected fastq files for m6A MeRIP sequencing (PRJNA927821) were obtained from METTL3 knockout and control samples^22^. Bowtie2 was used to align reads to the mouse reference genome. The mapped reads of the IP and input libraries were provided for MACS2 peak calling (https://pypi.org/project/MACS2/2.2.6/), a tool that identifies m6A peaks in bed or bigwig format, which can be visualized using Integrative genomics viewer (IGV) software (http://www.igv.org). To discover both unknown and known motifs, MEME (http://meme-suite.org) was used, followed by motif localization with respect to the peak summit. The called peaks were annotated by intersecting them with the gene architecture using R package ChIPseeker (Version 1.38.0). Subsequently, StringTie (https://ccb.jhu.edu/software/stringtie) was used to evaluate the expression of all mRNAs in the input libraries by calculating (fragments per kilobase of transcript per million mapped reads, multiplied by the exon length in kilobases). Differentially expressed mRNAs were selected based on log2|fold change| ≥ 1 and p < 0.05 using the limma package.

### Spatial transcriptome sequencing analysis

The datasets for spatial transcriptomics (ST) of colorectal cancer were obtained from the Sequence Read Archive (SRA) PRJNA942633. To capture gene expression information on ST slides, the Visium Spatial platform using 10× Genomics was used^23^. This involved the use of spatially barcoded RNA oligonucleotides according to the default protocol. The quality of the raw sequencing reads from the ST was assessed and aligned using Space Ranger (version 2.0.0). The resulting gene-spot matrices obtained from processing ST data and Visium samples were analyzed using the Seurat package (version 4.3.0) in R. Loci with a minimum threshold of 200 genes were selectively selected, whereas genes with fewer than eight read counts or fewer than four loci were ignored. Point alignment was achieved by implementing normalized LogVMR functions. For dimensionality reduction and clustering, principal component analysis (PCA) was employed with a resolution of 2.0, resulting in the identification of 23 distinct clusters, which was performed based on the scRNA-seq or ST signature using the default parameters in Seurat. The SpatialFeaturePlot function in Seurat was used to depict the spatial feature expression.

### m6A dot blot assay

Total RNA was extracted using TRIzol reagent, and mRNA was purified using the Dynabeads mRNA Purification Kit (Invitrogen). The concentration and purity of the mRNA were determined using a NanoPhotometer Spectrophotometer. Subsequently, the mRNA was denatured by heating at 95 °C for 5 min, followed by rapid cooling on ice. Next, mRNA, ranging from 200-400 ng of, was strategically placed onto the Amersham Hybond-N+ membrane (RPN303B, Cytiva) and delicately air-dried for 5 min. To establish covalent bonding, the membrane encountered a 254 nm UV crosslinking process in an Ultraviolet Crosslinker (254 nm UV for 5 min); subsequently to this, it was blocked with 5% skim milk in TBST and incubated overnight at 4 °C with the anti-m6A antibody (Proteintech). For further immunodetection, HRP-conjugated anti-mouse IgG secondary antibody was introduced onto the membrane and incubated at room temperature for 1 h. Accurate signal visualization was achieved by chemiluminescence. Methylene blue (0.02%; Sigma-Aldrich) was used to ensure equal distribution of RNA content across the entire membrane.

### Chromatin Cleavage Under Targets and Release Using Nuclease (Cut&Run)-qPCR

Chromatin coprecipitation analysis was conducted using an anti-β-catenin antibody (8480, Cell Signaling Technology), following the guidelines stipulated in the Cut&Run Assay Kit (HD101, Vazyme), as provided by the manufacturer. Fold enrichment was determined using qRT-PCR and expressed as a proportion of the input chromatin (percentage of input). Detailed information regarding the primers used is provided in Table S2.

### Chromatin immunoprecipitation (ChIP) sequencing analysis

We downloaded fastq files specifically chosen for β-catenin ChIP-seq (PRJNA777812) from SRA (https://www.ncbi.nlm.nih.gov/sra/). Bowtie2 was used to align reads to the reference genome. The aligned reads from both the IP and input libraries were utilized for MACS2 peak calling, an approach that identifies β-catenin peaks on chromatin in either bed or bigwig format, allowing for smooth visualization using IGV software. To discover both de novo and known motifs, MEME was applied, followed by localization of the identified motif in relation to the summit of each peak. The called peaks were annotated by intersecting them with the gene architecture using the R package ChIPseeker.

### Polysome profiling

HCT8-NC and HCT8-circMVP cells were cultured in a 10 cm dish until they reached approximately 80% confluence, and were subsequently employed for polysome profiling. Prior to collection, CHX (C4859; Sigma-Aldrich) was introduced into the medium at a concentration of 100 mg/ml for 7 min to inhibit active mRNA translation. Afterward, the medium was aspirated and the cells were rinsed thrice with precooled 4 °C PBS (containing 100 mg/ml CHX). Following centrifugation at 500×g for 5 min, the cell pellets were resuspended in 500 ml of lysis buffer (containing 20 mM HEPES pH 7.6, 100 mM KCl, 5 mM MgCl2, 100 mg/ml CHX, 1% Triton X-100, 1% protease inhibitor cocktail, and 40 U/ml RNase inhibitor), and the lysate was maintained on ice for 30 min. Subsequently, the lysate was subjected to centrifugation at 15,000 g for 15 min at 4 °C. Afterwards, 400 ml of supernatant was layered onto a sucrose gradient ranging from 10% to 50% (A502792, Sangon), which contained 20 mM HEPES pH 7.6, 100 mM KCl, 5 mM MgCl2, 100 mg/ml CHX, as well as protease and RNase inhibitors (B6004782, Sangon). The gradient was then subjected to ultracentrifugation at 45,000 rpm for 2 h at 4 °C, utilizing Beckman Coulter L-100XP Ultracentrifuge. The resulting sample was separated into 20 fractions using a fraction collector system (ASTM5307, Shimadzu). The absorbance at A260 nm was recorded for each fraction, and a portion of each fraction was subjected to western blotting. RNA was extracted from the fractions using TRIzol reagent, followed by RT-qPCR analysis to evaluate gene mRNA expression.

### Statistical analysis

Statistical analyses were conducted using GraphPad Prism 8.0 and SPSS (V22.0, IBM). The data obtained from western blotting and IF were compared using ordinary one-way analysis of variance (ANOVA), with multiple comparisons included when necessary. The protein expression results among the different groups are presented in quantification bar graphs, detailing the means accompanied by their respective standard deviations. The hazard ratio (HR) was determined using the Cox proportional hazards model, with a reported confidence interval of 95%. Additionally, Kaplan-Meier survival curve was generated. The association between circMVP expression, METTL3, B7-H3 expression, and clinicopathological parameters was examined using Pearson's χ2 test or Fisher's exact test. The comparison of mouse models among different groups was performed using the t-test and one-way ANOVA analysis, with multiple comparisons included when necessary. The results are expressed as the mean ± SD, and statistical significance was set at p < 0.05.

### Data availability

All data needed to evaluate the conclusions in the paper are presented in the paper and/or Supplementary Materials. High-throughput raw and processed data were deposited in the SRA.

## Results

### High expression of circMVP in CRC

**Figure 1:**
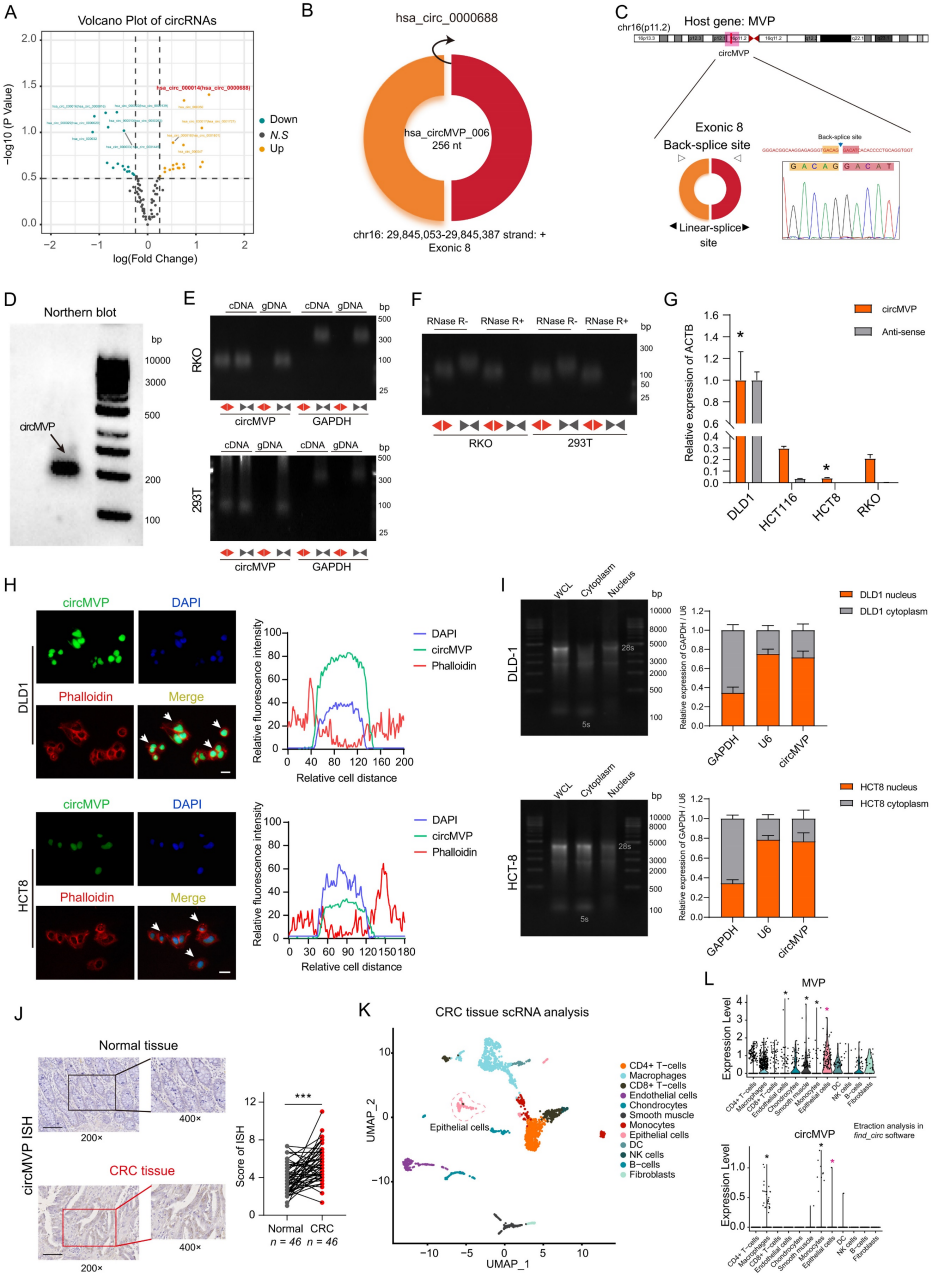
** Identification of circMVP expression in CRC. (A)** Volcano plot of differentially expressed circRNAs from transcriptome analysis of CRC tissues (*n=3*) and paired adjacent normal tissues (*n=3*). **(B)** Diagram of the structure and site information of hsa_circ_0000688 (circMVP) molecule formation. **(C)** Circular RNA formation patterns and Sanger sequencing of back-splicing regions of circMVP. **(D)** Expression of circMVP detected by Northern blot analysis. **(E)** Combination of PCR with an electrophoresis assay indicating the presence of circMVP using divergent and convergent primers from cDNA or genomic DNA (gDNA) in RKO and 293T cells. **(F)** Expression of circMVP and liner MVP in RKO and 293T cells cell lines detected by PCR assay followed by nucleic acid electrophoresis in the presence or absence of RNase R. **(G)** Expression of circMVP in CRC cell lines (DLD1, HCT8, HCT116, RKO) detected by qRT-PCR. **(H)** FISH analysis showing the localization of circMVP mainly in the nucleus and a small fraction in the cytoplasm (DLD1 and HCT8). Nucleus was stained blue with DAPI, cytoskeleton was stained with phalloidin (red) and circMVP was stained with FITC (green). Scale bar, 20 μm. **(I)** RNA of CRC cell lines (DLD1 and HCT8) detected by nucleoplasmic separation, gel electrophoresis and qRT-PCR, relative to the expression of GAPDH. **(J)** Expression of circMVP examined by ISH in normal epithelial tissues (n=46) and CRC tissues (n=46). Scale bar, 50 μm. **(K)** UMAP plot showing the clusters of scRNA-seq in CRC samples. Each dot is a cell colored by its analyzed cell types. **(L)** UMAP plot showing the distribution and expression of circMVP and its host gene. MVP. Results are presented as mean±SD. *p < 0.05; **p < 0.01; ***p < 0.001 (Student's *t*-test; log-rank test). CRC, colorectal cancer; ISH, *in situ* hybridization; UMAP, uniform manifold approximation and projection.

A total of 228 unique circRNA candidates were identified in the CRC samples. Among these, 19 circRNAs exhibited up-regulation, while 18 circRNAs were down-regulated in CRC tissues as compared to the paired normal tissues used as control (log2|fold change| ≥ 1 and *P* < 0.05; Fig. 1A, Table S4 and Fig. S1A-B). The discrepancy in circRNA expression between CRC and paired adjacent normal tissues was demonstrated using a scatter plot (Fig. 1A). CircMVP (hsa_circ_0000688, Alias_000014) was back-spliced at exon 8 of the MVP gene, with a spliced sequence length of 256 nt (Fig. 1B), and steadily upregulated in CRC tissues. Sanger sequencing also validated the arrangement of the head-to-tail fusion positions (Fig. 1C). Northern blotting analysis showed that circMVP was expressed in CRC cells (Fig. 1D).

The RKO (CRC cell) and 293T were used to investigate the presence of circMVP. PCR and agarose gel electrophoresis analyses were conducted using specifically designed divergent and convergent primers, revealing the presence of back-spliced or canonical forms of circMVP as opposed to linear MVP (Fig. 1E and Fig. S1B). The presence of circular MVP was confirmed through resistance to RNase R digestion (Fig. 1F). Thus, circMVP expression was assessed in CRC cell lines DLD1, HCT8, HCT116, and RKO. Among these cell lines, DLD1 and RKO cells exhibited the highest and lowest levels of circMVP expression, respectively (Fig. 1G). In addition, MVP expression was consistent with the trend of circMVP, and these expression characteristics were consistent with the biological process of circRNA generation (Fig. 1G, Fig. 1I, and Fig. S1C).

The RNA fluorescence *in situ* hybridization (FISH) assay revealed circMVP is mainly localized in the nucleus, although its presence in the cytoplasm was also observed (Fig. 1H). Nuclear and cytoplasmic isolation confirmed that circMVP was expressed mainly in the nucleus and partly in the cytoplasm (Fig. 1I and Fig. S1D). In the CRC samples, circMVP was assessed by situ hybridization (ISH) analysis of excised tissue sections. The results revealed elevated expression of circMVP in CRC tissues compared to that in normal epithelial tissue (Fig. 1J and Fig. S1E). The cellular composition of the immune microenvironment in CRC was evaluated by single-cell RNA sequencing. (scRNA-seq). Cellular composition was elucidated through impartial clustering of all cells via PCA and subsequently presented visually using UMAP, including the detection the cell types of CD4+ T cells, CD8+ T cells, macrophages, endothelial cells, Chondrocytes, Smooth muscle, Monocytes, Epithelial cells, dendritic cells, natural killer (NK) cells, B-cells and Fibroblasts (Fig. 1K). MVP and circMVP were mainly expressed in the epithelial cells (CRC cells) cluster (Fig. 1L and Fig. S1F). Therefore, we examined the specific expression of circMVP in CRC cells.

### CircMVP facilitates CRC growth *in vitro* and *in vivo*

**Figure 2:**
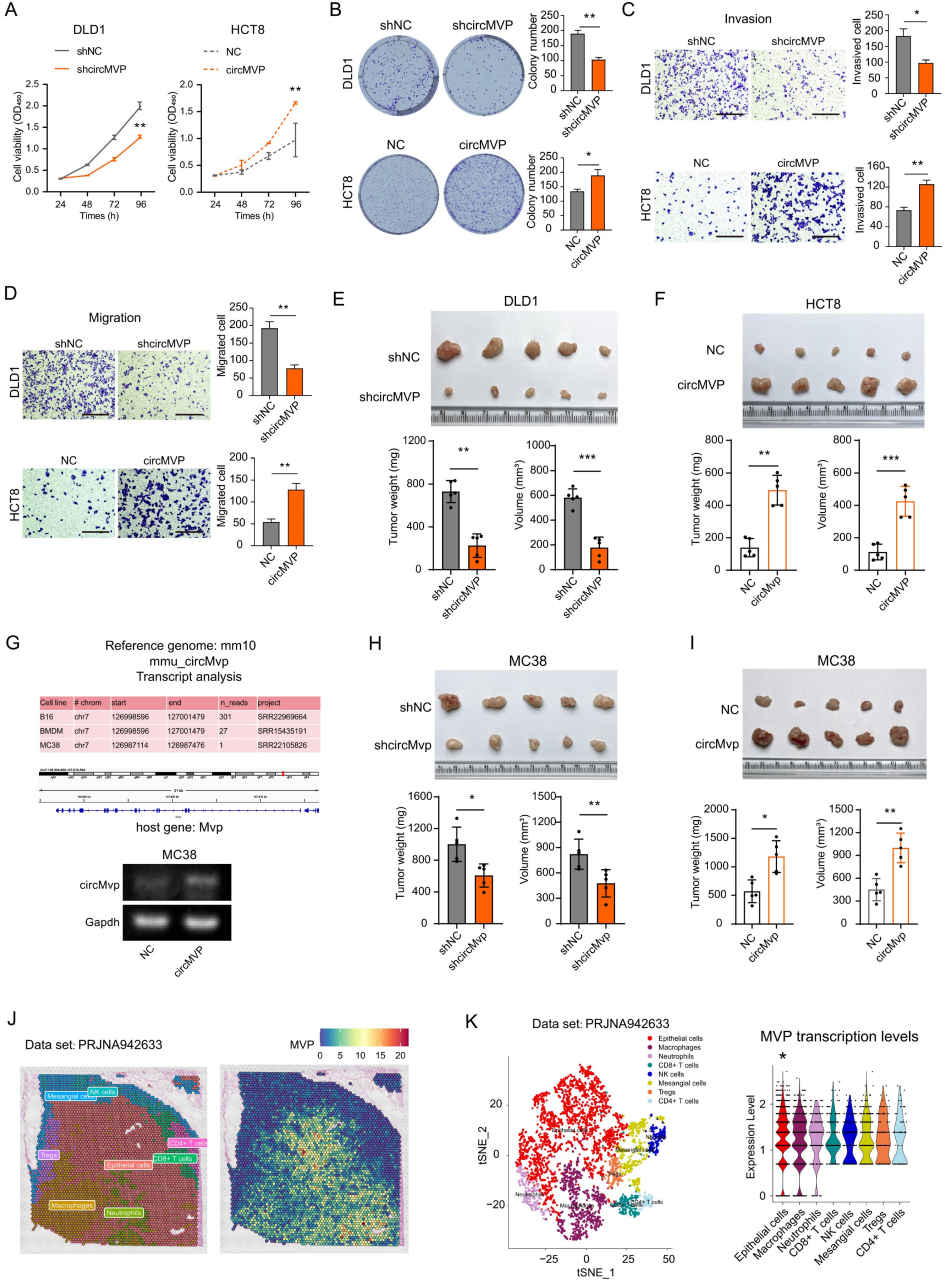
** circMVP facilitates CRC growth. (A and B)** Effect of circMVP on cell proliferation (A) and colony formation (B). **(C**) Transwell-matrigel was used to detect the effect of circMVP on the invasion ability of cancer cells. Scale bar, 300 μm. **(D)** Transwell was used to detect the effect of circMVP on the migration ability of cancer cells. Scale bar, 300 μm. **(E and F)** Effect of circMVP on CRC tumor growth *in vivo.* DLD1 (E) and HCT8 (F) were evaluated using a xenograft model. Tumor volume and weight were measured after mouse sacrifice (n = 5). **(G)** circMVP sequence was compared in mouse genome, circMVP overexpression and knockdown MC38 cells were constructed, and PCR identification was performed. **(H and I)** Effect of circMVP on CRC tumor growth *in vivo* in an MC38 model. Tumor volume and weight were measured after mouse sacrifice (n = 5). **(J and K)** Pathological section and TSNE plot showing the clusters of spatial transcriptome in the CRC samples from PRJNA942633. Each dot is a cell colored by its analyzed cell types. Violin plot showing MVP transcription levels and distribution among cell types. Results are presented as mean±SD. *p < 0.05; **p < 0.01; ***p < 0.001 (Student's *t*-test; log-rank test). CRC, colorectal cancer; TSNE, t-distributed stochastic neighbor embedding.

The endogenous expression of circMVP was measured in CRC cell lines, DLD1 and HCT8 cells with relatively high and low circMVP expression, and selected for subsequent functional assays for transfection efficiency (Supplementary Fig. S1G). CCK-8 and colony formation assays illustrated that depletion of circMVP markedly impeded CRC cell proliferation and colony formation, whereas overexpression of circMVP facilitated both these phenomena. (Fig. 2A, Fig. S2A and 2B). The impact of circMVP on the metastasis of CRC was further investigated through a transwell assay, which revealed that the depletion of circMVP repressed the migration and invasion of DLD1 and HCT8 cells. Conversely, overexpression of circMVP significantly stimulated these processes (Fig. 2C and 2D, Fig. S2B-S2F). Furthermore, circMVP silencing inhibited the tumorigenicity of CRC in mice, whereas circMVP overexpression promoted its tumorigenicity (Fig. 2E and 2F).

A comparison of the Homo sapiens genome with the mouse genome, and circMVP was found to be expressed in a variety of murine-derived cells, including colorectal cancer, further demonstrating the conserved and stable interspecies expression of circMVP. (Fig. 2G and Fig. S2G). The MC38 tumor model was established by subcutaneous inoculation of MC38 cells in C57BL/6 mice. circMVP facilitated the *in vitro* proliferation, colony formation, migration, and invasion of MC38 cells. Conversely, circMVP knockdown impeded the aforementioned processes (Fig. 2F-2H and Fig. S2H-S2I). Indeed, suppression of circMVP restrained the tumorigenic potential of CRC, whereas its overexpression stimulated it in C57BL/6 mice (Fig. 2H and Fig.2I). Furthermore, spatial transcriptome datasets of bowel cancer tissue samples were analyzed to evaluate the significantly higher levels of MVP transcription in epithelial cell communities (Fig. 2J, Fig. 2K and Fig. S2J). Collectively, these results suggest that circMVP has the function of promoting CRC progression.

### CircMVP stabilizes METTL3 by RNA binding protein

**Figure 3:**
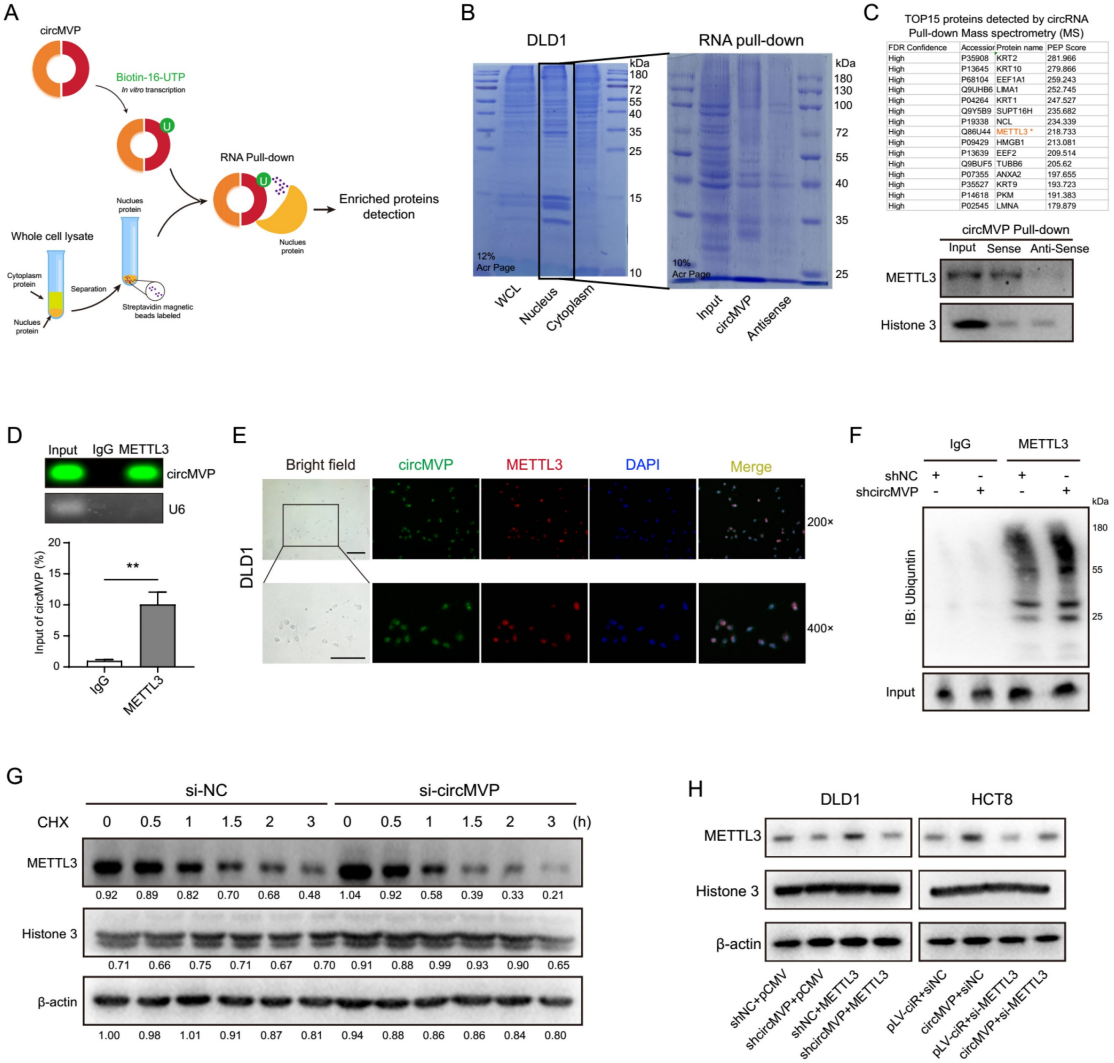
** circMVP directly interacts with METTL3. (A)** Schematic diagram of circMVP transcription *in vitro* binding to CRC nuclear protein for RNA pulldown experiment. **(B and C)** circMVP binding protein on SDS-page was stained with Coomassie blue (B), the level of circMVP binding to METTL3 was detected by MS analysis and western blot (C). **(D)** Level of METTL3 protein binding to circMVP detected by RIP and analyzed by gel electrophoresis and qRT-PCR. **(E)** FISH-IF detected circMVP binding and colocalization with METTL3, circMVP (FITC), METTL3 (Cy3), and DAPI labeled nucleus (DAPI). Scale bar, 20 μm. **(F)** CircMVP expression inhibited the ubiquitin modification of METTL1. DLD1 cells were transfected with si-circMVP or METTL3 and treated with MG132 (20 mmol/mL) for 3 hours, the binding ubiquntin level was detected. **(G)** Effect of circMVP on the endogenous METTL3 protein expression in CRC cells treated with CHX (50 µg/mL), Quantification of the gray bands was performed using ImageJ. **(H)** Effect of circMVP on METTL3 protein expression in CRC cells detected by western blot. Results are presented as mean±SD. *p < 0.05; **p < 0.01; ***p < 0.001 (Student's *t*-test; log-rank test). RIP, RNA immunocoprecipitation; FISH, fluorescence *in situ* hybridization; IF, immunofluorescence; EMSA, electrophoretic mobility shift assay.

CircMVP transcription was labeled with biotin *in vitro* to explore downstream molecules. According to previous results, circMVP is mainly expressed in the nucleus of CRC cells. Therefore, nuclear proteins were used to label streptavidin magnetic beads for RNA pull-down detection (Fig. 3A). The proteins binding to circMVP were identified by Coomassie blue staining. Proteins Mass Spectrometry analysis of specific bands and western blot analysis, revealing that circMVP directly bound to the METTL3 protein (Fig. 3B, 3C and Fig. S3A, Table S5). CircMVP binding to METTL3 was further confirmed by RNA immunoprecipitation (RIP) detection, which revealed the RIP of METTL3 with circMVP (Fig. 3D). The RNA electrophoretic mobility shift assay (EMSA) confirmed that the nucleic acid of circMVP bound specifically to the METTL3 (Fig. S3B). IF and FISH detection in DLD1 cells demonstrated that circMVP and METTL3 mainly colocalization in the nucleus (Fig. 3E). Moreover, we observed that inhibiting proteasome activity prevented si-circMVP-induced endogenous METTL3 downregulation in DLD1 cells, suggesting that METTL3 degradation via the ubiquitin is inhibited by circMVP (Fig. 3F and Fig. S3C).

Cycloheximide (CHX), inhibitor of protein synthesis, was used to assess the impact of circMVP on the degradation process of METTL3. CircMVP knockdown in DLD1 cells prolonged the degradation of METTL3 (Fig. 3G and S3D). METTL3 functions as a pivotal catalyst within the MTase complex, aiding the incorporation of m6A modifications. Therefore, the effect of METTL3 on circMVP expression was assessed. The RNA stability assay revealed that circMVP stability increased after binding to METTL3 (Fig. S3E-H). The METTL3 plasmid and siMETTL3 were transferred for circMVP overexpression and knockdown, respectively, indicating the regulatory effect of circMVP on METTL3 (Fig. 3H). These results suggest that circMVP binds to and stabilizes METTL3 expression, thereby exerting a downstream potential to promote CRC progression.

### CircMVP facilitates METTL3-m6A modification strengthening β-catenin by targeting CTNNB1

**Figure 4:**
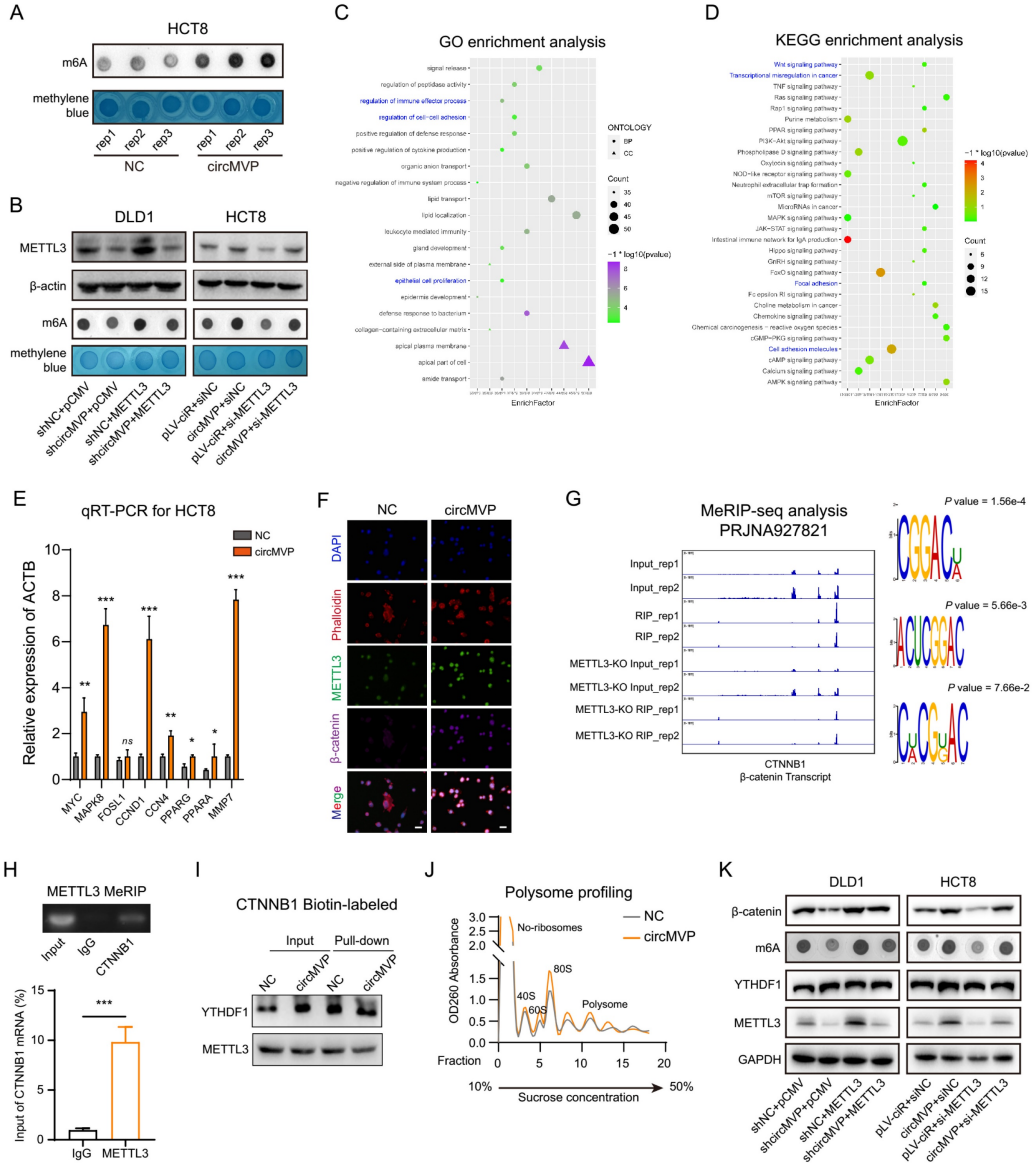
** circMVP facilitates METTL3-m6A modification in CTNNB1 mRNA. (A)** The level of RNA m6A modification of circMVP in HCT8 detected by dot blot. **(B)** Effect of circMVP on METTL3 protein expression in CRC cells detected by western blot and dot blot. **(C and D)** Gene ontology (GO) and KEGG pathway enrichment analysis terms of the DEGs in HCT8-circMVP compared with HCT8-NC. **(E)** RNAseq data analyzed transcription levels of downstream marker molecules activated by Wnt/β-catenin signaling pathway, relative to β-actin expression. **(F)** IF analysis showing that METTL3 and β-catenin protein expression and localization. Nucleus was stained in blue by DAPI, cytoskeleton was stained with phalloidin (red), METTL3 was stained with FITC (green) and β-catenin was stained with Violet 395 (purple). Scale bar, 20 μm. **(G)** Integrative genomics viewer (IGV) tracks showing m6A peaks distribution on CTNNB1 transcript from MeRIP-seq data in PRJNA927821. **(H)** RIP in PCR and qRT-PCR was used to detect the relative mRNA level of CTNNB1 in DLD1 (n = 3). **(I)** Western blotting analysis of YTHDF1 after RNA pull-down assay with biotinylated-CTNNB1, in cell lysates of HCT8-circMVP compared with HCT8-NC. **(J)** Polysome profiling of HCT8-NC and HCT8-circMVP cells. **(K)** Effect of circMVP on METTL3 and β-catenin protein expression in CRC cells detected by western blot and dot blot. Results are presented as mean±SD. *p < 0.05; **p < 0.01; ***p < 0.001 (Student's *t*-test; log-rank test). m6A, N6-methyladenosine; FISH, fluorescence *in situ* hybridization; IF, immunofluorescence; KEGG, Kyoto Encyclopedia of Genes and Genomes.

METTL3 functions as a catalytic nucleus within the MTase complex, orchestrating the incorporation of m6A^24^. Assessment of the biological function of circMVP-mediated METTL3 regulation revealed that circMVP upregulated RNA m6A expression, as detected by dot blotting (Fig. 4A). When circMVP or METTL3 was knocked down, the expression of METTL3 and m6A decreased (Fig. 4B). In contrast, the expression of METTL3 and m6A increased after upregulation of circMVP or METTL3 (Fig. 4B). Next, transcriptomic sequencing of the constructed circMVP that was stably overexpressed in HCT8 cells was performed (Fig. S4A). GO analysis revealed that circMVP activated biological processes, including Immune effector processes, Intercellular adhesion, and Proliferation of epithelial cells (Fig. 4C). KEGG analysis suggested that circMVP promoted the activation of the Wnt signaling pathway, transcriptional dysregulation in cancer, focal adhesion, and cell adhesion molecules (Fig. 4D and Fig. S4B). GSEA suggested that circMVP promoted Wnt/β-catenin signaling activation (Fig. S4C). In addition, the gene transcription levels regulated by Wnt/β-catenin signaling pathway activation were assessed. Downstream gene clusters regulated by β-catenin were significantly up-regulated following circMVP overexpression (Fig. 4E). IF analysis of circMVP, METTL3 and β-catenin expression and localization in cells revealed that circMVP overexpression stabilized METTL3, and up-regulated β-catenin expression and nuclear ectopia. In addition, it is suggested that circMVP/METTL3 may regulate downstream signaling pathways and transcriptional activation through β-catenin signaling (Fig. 4F).

Modification of m6A is involved in various molecular processes in mRNA metabolism, encompassing alternative splicing, precise translation, and fine-tuned regulation of mRNA stability^25^. This study aimed to explore the impact of METTL3-mediated m6A modification by analyzing the distribution of m6A modification sites with substantial certainty within the RNA sequence of CTNNB1, utilizing a sequence-based predictor of RNA adenosine methylation. Both human and murine CTNNB1 mRNA had high confidence intervals for m6A modification (Fig. S4D and Fig. S4E). We performed an analysis of m6A methylated immunoprecipitation RNA sequencing (MeRIP-seq), and IGV demonstrated a reduction in m6A peaks on CTNNB1 transcripts in METTL3-KO. Moreover, motif enrichment analysis revealed a shared sequence motif among the aforementioned m6A peaks "GGACU"(Fig. 4G); thus, the effect of METTL3-m6A modifications on the CTNNB1 transcript was investigated. At the molecular level, m6A modification exerts its influence in the regulation of multiple mRNAs metabolism. To elucidate the effect of METTL3-mediated m6A modification on CTNNB1 mRNA metabolism, PCR-Agarose gel electrophoresis was used to check CTNNB1 transcription for qualitative evaluation, and qRT-PCR was used to quantitatively analyze METTL3 binding CTNNB1 mRNA (Fig. 4H). In addition, in mouse tumor cell lines Ctnnb1 (murine gene symbol for β-catenin) was stably expressed, and MC38 cells were subsequently used as *in vivo* model in this work (Fig. S4F). These results suggest that circMVP/METTL3 catalyzes β-catenin signaling and regulates transcriptional activation of its downstream genes.

YTHDF1 is a key m6A reading protein that promotes m6A deposition and mRNA translation efficiency through interactions with initiation factors and ribosomes^26^. YTHDF1 involvement in the m6A "Reader" effect of METTL3 on CTNNB1 mRNA was investigated. RIP-qPCR and streptavidin RNA pull-down assay revealed that YTHDF1-mediated augmentation of β-catenin, both endogenously and exogenously, and there was no significant change in CTNNB1 transcription in DLD1 cells (Fig. 4I and Fig. S4G-H). The qRT-PCR results indicated that circMVP/METTL3 did not affect CTNNB1 transcription (Fig. S4I). Subsequently, various RNA fractions from the control group (NC) and circMVP were isolated using ribosome profiling. The results demonstrated that CTNNB1 mRNA expression in translationally active polysomes (>80S) of NC cells was significantly lower than that in circMVP cells, whereas the expression in monosomes (<40S, 40S, 60S, and 80S) was not significantly different (Fig. 4J). Remarkably, restoration of m6A and β-catenin protein expression was observed upon overexpression of METTL3 in DLD1 cells and downregulation of METTL3 in HCT8 cells (Fig. 4K).

### CircMVP promotes METTL3-activated B7-H3 dependent immunosuppression of TME in CRC

**Figure 5:**
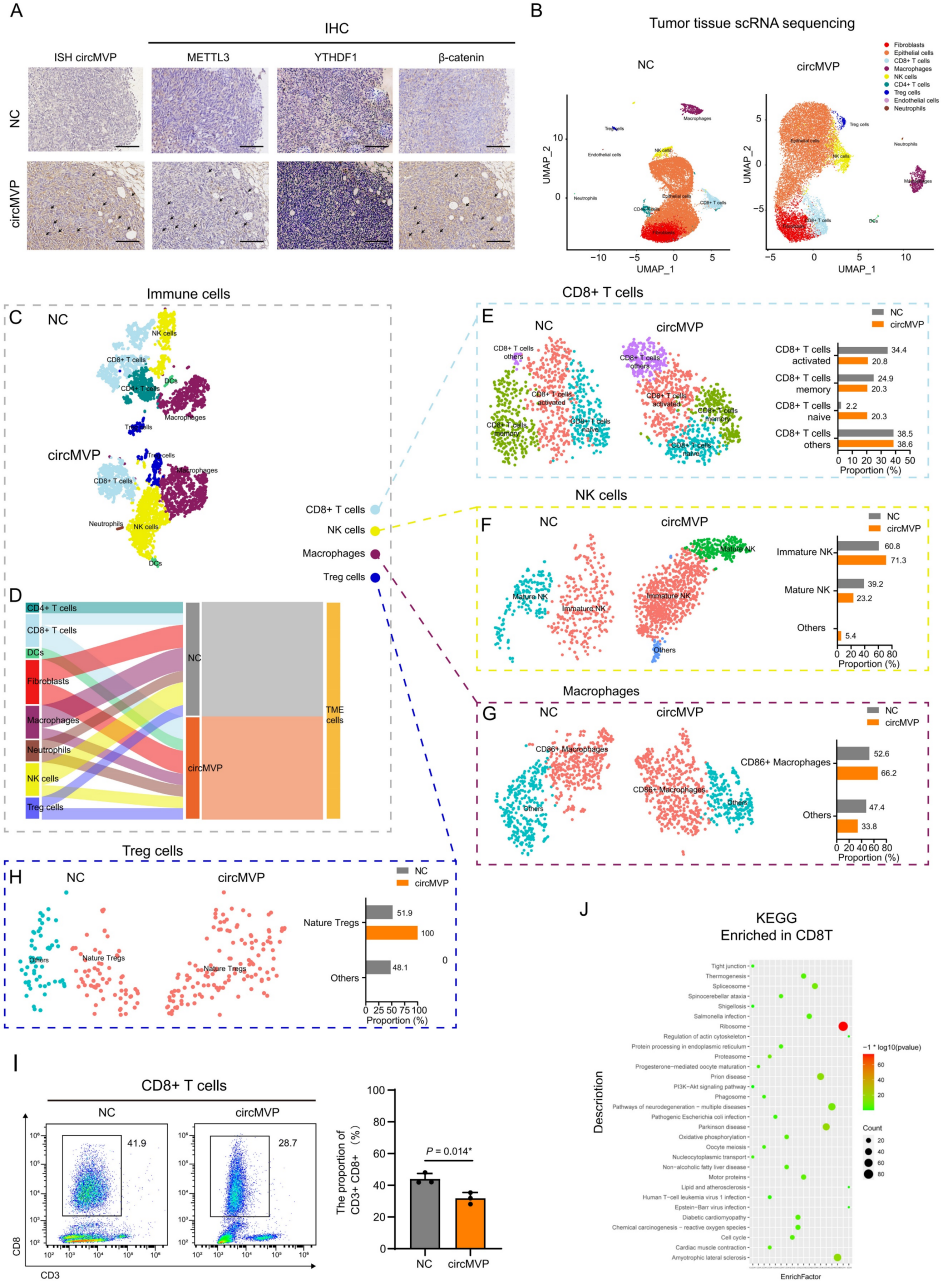
** circMVP promotes METTL3 activated immunosuppression. (A)** Expression of circMVP in the tumor of MC38 CRC mouse model (NC *vs.* circMVP) detected by ISH, and the expression of METTL3, YTHDF1 and β-catenin detected by IHC, Scale bar, 100 μm. **(B)** UMAP plot showing the cell types of scRNA-seq in the tumor of MC38 CRC mouse model (NC *vs*. circMVP). Each dot is a cell colored by its analyzed cell types.** (C)** Feature plots of characteristic markers of epithelial cell types showing the low expression in dark red and high expression in bright red. **(D)** Sankey plot showing cell type of TME in MC38-NC and MC38-circMVP. **(E-H)** scRNA sequencing analysis. Expression of CD276 in MC38-circMVP compared with that of MC38-NC, including CD8+ T cells (E), NK cells (F), Macrophages (G) and Tregs (H). **(I)** Flow cytometry analysis of CD8+ T cell infiltration in the tumor of MC38-NC and MC38-circMVP; scatter plot on the left, statistical histogram on the right. **(J)** KEGG pathway enrichment terms of the DEGs in CD8+ T cells of MC38-circMVP compared with CD8+ T cells of MC38-NC. Results are presented as mean±SD. *p < 0.05; **p < 0.01; ***p < 0.001 (Student's *t*-test; log-rank test). CRC, colorectal cancer; TSNE, t-distributed stochastic neighbor embedding. ISH, *in situ* hybridization; IHC, immunohistochemistry; UMAP, uniform manifold approximation and projection.

The role of circMVP in the regulation of anti-tumor immunity was investigated by transfecting MC38 murine CRC cells with circMVP using lentivirus and subsequently injecting these cells into syngeneic C57BL6 mice. In the circMVP group compared to the NC group, enhanced expression of circMVP, METTL3, YTHDF1 and β-catenin was observed, as detected by ISH and IHC techniques (Fig. 5A). Furthermore, an increase in the percentage of cells exhibiting β-catenin nuclear localization was observed (Fig. 5A). The impact of circMVP on CRC and TME was explored by isolating and collecting a single-cell suspension from a tumor and scRNA-seq was performed (NC: 18294 cells; circMVP: 15040 cells). Tumors with circMVP T and NK cells were significantly increased in the NC group (Fig. 5B and Fig. S5A). Epithelial cells, CD4+ T cells, CD8+ T cells, Macrophages, Treg cells, endothelial cells, Neutrophils and NK cell subsets were further clustered, and were simultaneously increased in circMVP overexpression group compared to control (Fig. 5B). We hypothesized that circMVP suppresses anti-tumor immunity by inducing negative stimulating molecules in cells. The functional markers of epithelial cells, Grb2 and Krt14, were examined, and these genes were mainly enriched in epithelial clusters (Fig. S5B). The effect of circMVP on the TME in CRC tumorigenesis was investigated by analyzing TME cells in colorectal tissue. Clustering analysis showed a significant expansion of CD45+ immune cells, CD4+ T cells, CD8+ T cells, macrophages, neutrophils, NK cells, and DCs, and a contraction of fibroblasts in the circMVP group compared to the NC group (Fig. 5C and Fig. 5D). CD8+ T cells are the main anti-tumor immune effector cells, and CD8+ T cells are the important cellular components of tumors and are involved in the field of anti-tumor immunity in a complex way. This study explored the effect of circMVP on immune cell populations, subcluster analysis showed that the proportion of activated CD8+ T cells decreased in the circMVP group compared with the NC group, while the proportion of initial CD8+ T cells increased (Fig. 5E). We also evaluated the effect of circMVP on NK cells. Mature, immature, and other NK cell subtypes are identified based on their genetic markers. An increase in the proportion of immature NK cells and a decrease in the proportion of mature NK cells were observed in the circMVP group. Other types of NK cells were also found in the circMVP group, but their numbers were too small to be accurately identified (Fig. 5F). The impact of circMVP on macrophages was assessed by the identification of two subclusters: CD86+ macrophages and others that were not identified (Fig. 5G). In addition, under the action of circMVP, Treg cell clusters further divided into natural Treg cells; However, due to reduced numbers, it is not possible to identify other communities as definite Tregs subgroups (Fig. 5H).

The above results suggested that circMVP might interfere with adaptive immunity in CRC by affecting T cells. The expression of immune checkpoint markers was analyzed in tumor (epithelial) cells, and this hypothesis was supported by the altered transcription levels of costimulatory molecules in the circMVP group (Fig. S5C and Fig. S5D). The proportion of CD8+ T cells in the tumor affected by circMVP was confirmed using flow cytometry of immune cells. CD8+ T cells in the circMVP-overexpressing group were significantly lower than those in the NC group (Fig. 5I). CD8+ T cells labeled by NC and circMVP4 were analyzed, and the differentially expressed genes in CD8+ T cells of the circMVP and NC groups suggested that circMVP inhibited the activation of the CD8+ T immune response (Fig. 5J and Fig. S5E-H). Thus, the results from *in vivo* data support the immunosuppressive function of circMVP in CRC.

### CircMVP/METTL3 transcriptionally regulates B7-H3 expression by β-catenin

**Figure 6:**
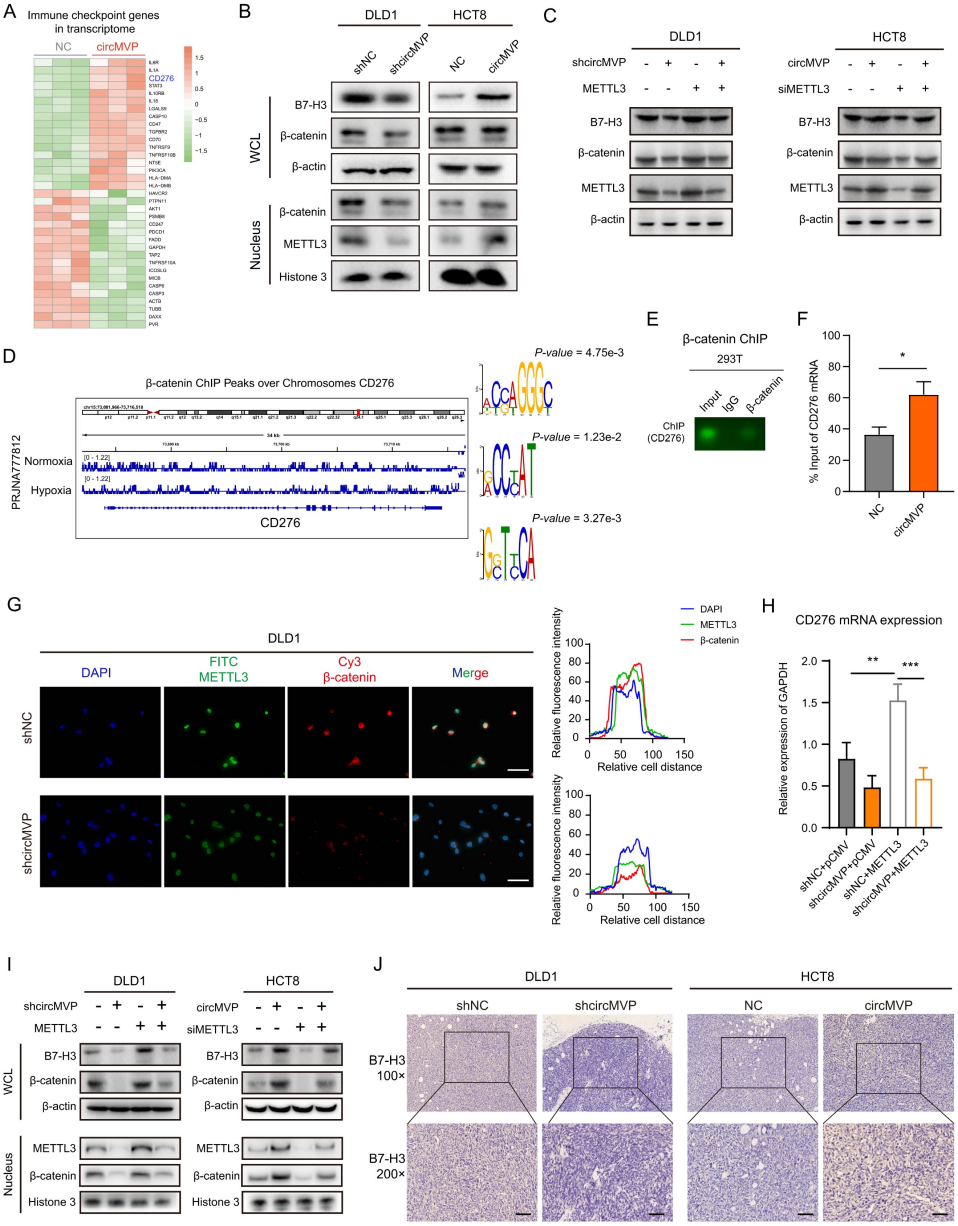
** circMVP/METTL3/β-catenin regulates B7-H3 expression.** Transcriptome analysis of differentially expressed immune checkpoint genes in HCT8-circMVP compared with HCT8-NC. **(B)** Expression of β-catenin and B7-H3 regulated by circMVP detected by western blot after nuclear cytoplasmic separation. **(C)** β-catenin and B7-H3 protein expression regulated by circMVP/METTL3 detected by western blot. **(D)** Integrative genomics viewer (IGV) tracks showing β-catenin peaks distributed on B7-H3 (CD276) transcript from ChIP-seq data in PRJNA777812. **(E and F)** β-catenin in ChIP PCR and qPCR used to analyze the relative chromatin promoter region of CD276 in DLD1 (n = 3). **(G)** IF analysis of circMVP expression and localization of METTL3 (green) and β-catenin (red). **(H)** CD276 expression regulated by circMVP/METTL3 detected by qRT-PCR. **(I)** β-catenin and B7-H3 protein expression regulated by circMVP/METTL3 detected by separation of nucleus and cytoplasm and western blot. **(J)** CircMVP regulated B7-H3 expression detected by IHC. Scale bar, 60 μm. RIP, RNA immunocoprecipitation; ChIP, chromatin immunoprecipitation; IF, immunofluorescence; IHC, immunohistochemistry.

TcircMVP regulates the anti-tumor immune function of β-catenin by enhancing METTL3. The specific regulatory mechanism of circMVP needs further evaluation. Previous studies have shown that β-catenin in tumor cells is involved in gene regulation as a cotranscription factor, including regulating the expression of immune checkpoint genes. Transcriptome analysis showed that the transcription of CD276 (encoding B7-H3) was significantly upregulated (Fig. 6A and Fig. S6A). Nucleoplasmic separation results showed that the expression of METTL3, β-catenin and B7H3 proteins in DLD1 cells decreased after circMVP knockdown. After circMVP overexpression, METTL3 expression was increased, β-catenin nuclear signal was enhanced, and B7-H3 expression was up-regulated (Fig. 6B). In knockdown circMVP DLD1 cells, METTL3 overexpression significantly reversed β-catenin and B7-H3 proteins, while in HCT8 cells, circMVP was overexpressed and METTL3 was down-regulated, B7-H3 was also reversed. (Fig. 6C).

Next, the regulation of B7-H3 mediated by circMVP via METTL3/β-catenin was studied. ChIP-seq data analysis showed that β-catenin acted on the chromatin region of CD276, and motif analysis of IGV and MEME was performed (Fig. 6D and Fig. S4D). Both ChIP PCR-Agarose gel electrophoresis and ChIP qPCR analysis showed that β-catenin acted on the promoter region of CD276, thereby regulating the increase of CD276 transcription (Fig. 6E and 6F). IF detection showed that DLD1 cells with knocked down circMVP, METTL3, and β-catenin nuclear etopic decreased in circMVP cells (Fig. 6G). The knockdown of circMVP may inhibit the function of β-catenin as a transcription factor, qPCR showed that circMVP and METTL3 up-regulated the expression of CD276 (Fig. 6H). At the protein expression level, METTL3 was overexpressed in DLD1 cells with stable circMVP overexpression, and METTL3 was knockdown in HCT8 cells with stable circMVP overexpression, and then nuclear plasma separation detection showed that circMVP promoted the nuclear localization of β-catenin and the increase of B7-H3 protein. METTL3 knockout inhibited this phenomenon (Fig. 6I). IHC of nude mouse tumors showed that B7-H3 protein expression was decreased by circMVP knockdown, B7-H3 was mainly expressed in the cytoplasm and membrane of CRC cells, whereas circMVP expression was upregulated (Fig. 6J and and Fig. S6B). In order to explore whether METTL3 directly acts on CD276 mRNA and thus affects the expression of B7-H3 protein, we analyzed the METTL3 methylated RIPseq data set and found that there was no significant peak of METTL3 action in CD276 (Fig. S6C). The above results suggest that circMVP might regulate B7-H3 expression by promoting β-catenin to participate in transcriptional regulation through the binding of METTL3.

### Targeting circMVP enhances the efficacy of B7-H3 immunotherapy

**Figure 7:**
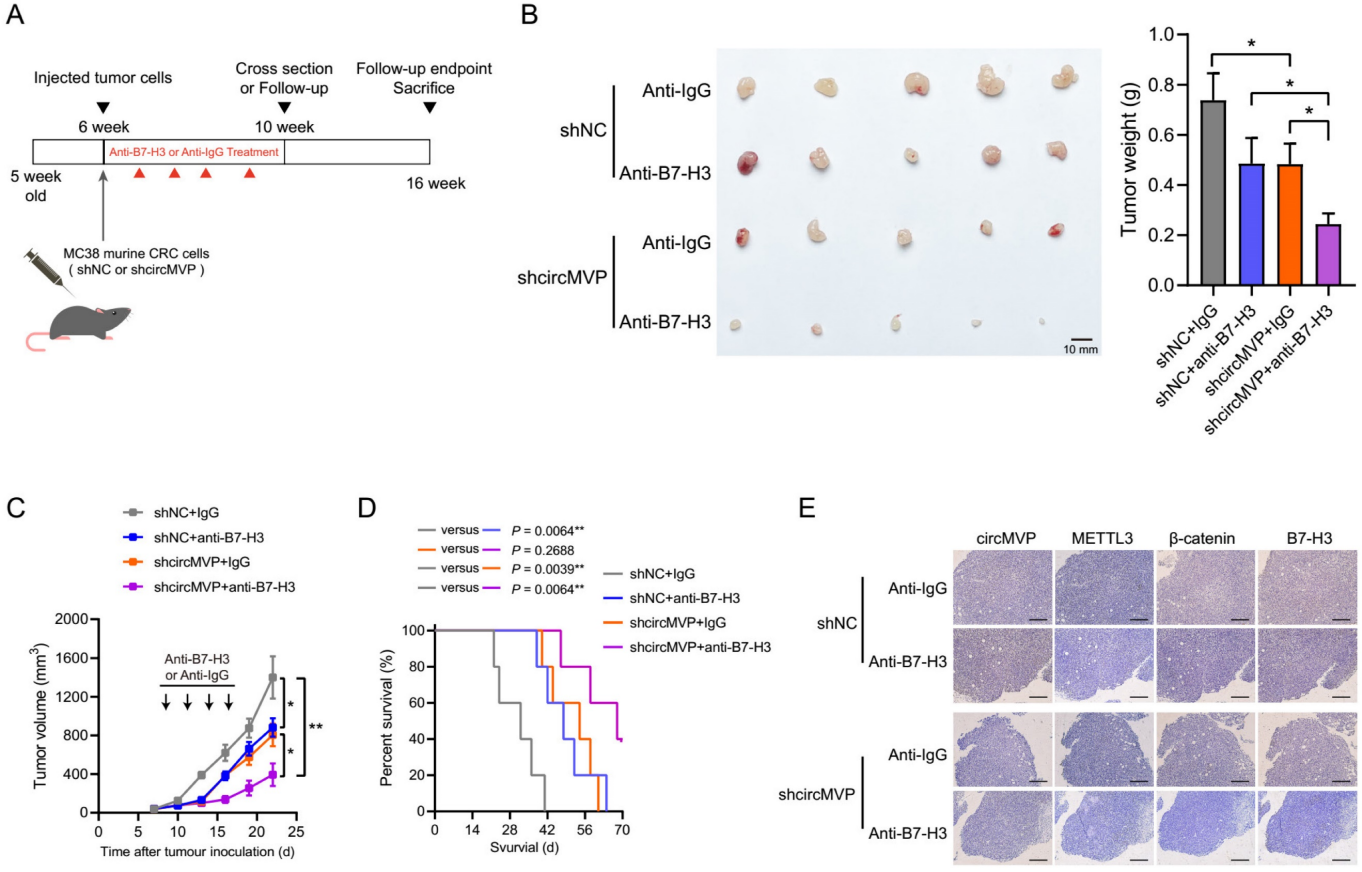
** circMVP affects tumor immune-efficacy through B7-H3. (A)** Scheme of circMVP and anti-B7-H3 treatment in colon cancer model. **(B)** Tumor growth in c57BL/6 mice carrying circMVP or control MC38 subcutaneous tumors, n = 6 per group. Intratumoral injection of anti-B7-H3 or IgG started on day 9. **(C)** Tumor volume of c57BL/6 mice carrying circMVP or control MC38 tumors, n = 6 per group. **(D)** Survival of c57BL/6 mice carrying circMVP or control MC38 tumors. **(E)** Representative circMVP expression detected by ISH, and METTL3, β-catenin and B7-H3 detected by IHC. Scale bar, 60 μm. Results are presented as mean±SD. *p < 0.05; **p < 0.01; ***p < 0.001 (Student's *t*-test; log-rank test; ANOVA or Fisher's exact tests). IHC, immunohistochemistry.

CircMVP promoted tumor production and exhibits inhibitory function of T cell effect (Fig. 5J and Fig. S5E). Importantly, it might suggest that CRC has an impact on ICB treatment susceptibility. Consequently, we propose to hypothesize that interventions targeting circMVP production or function may potentially enhance the therapeutic effect of ICBs, particularly in tumors that are circMVP positive and resistant to ICB. To validate this hypothesis, we treated ICB-resistant MC38 tumors with either the anti-B7-H3 antibody Omburtamab or a combination of circMVP negative control (NC) and knockdown (shRNA) (Fig. 7A). Consistent with our previous findings, knockdown circMVP decreased tumor growth (Fig. 7B and 7C). Moreover, the tumor growth in the anti-B7-H3 group was significantly lower than that in the anti-Ig group. It is worth noting that mice treated with anti-B7-H3 combined with circMVP knockdown showed significant tumor inhibition (Fig. 7B and 7C). Although the efficacy of monotherapies was somewhat satisfactory, the amalgamation of circMVP silencing with ICB resulted in a notable decrease in tumor burden, whereas the combination of circMVP+anti-B7-H3 remarkably ameliorated the overall survival rate (Fig. 7D). ISH and IHC detection of circMVP knockdown in tumors also found that it reduced β-catenin expression and nuclear ectopic, while downregulating the expression of B7-H3 (Fig. 7E). These findings provide key evidence that circMVP may inhibit the antitumor effects of T cells and the ICB synergies of B7-H3.

### CircMVP is clinically associated with METTL3 and B7-H3 in CRC

**Figure 8:**
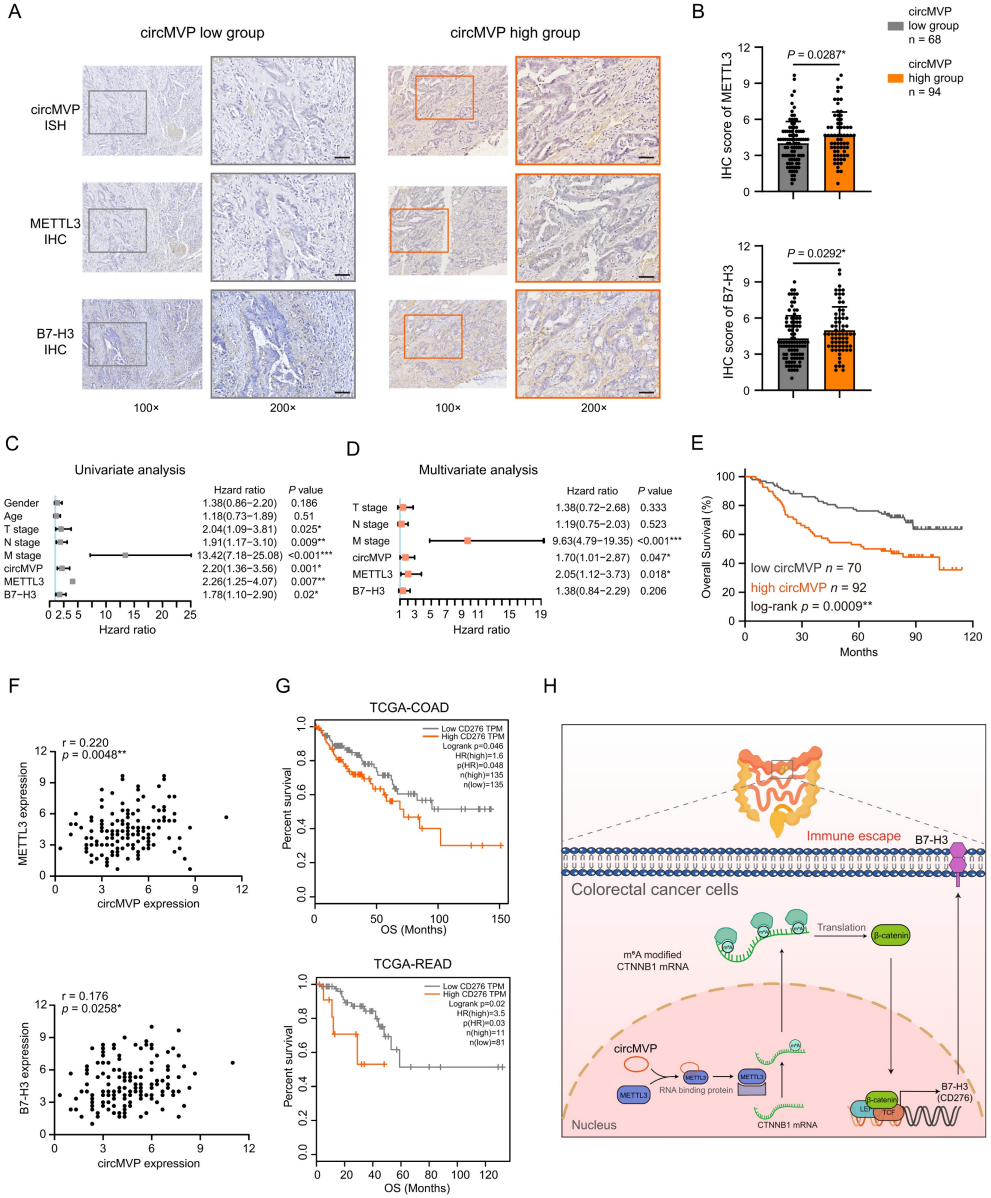
** CircMVP, METTL3 and B7-H3 affect the prognosis of CRC patients. (A and B)** Expression of circMVP in CRC tissue detected by ISH in high expression group (n=94) and low expression group (n=68). Expression of METTL3 and B7-H3 proteins detected by IHC. Scale bar, 60 μm. High expression circMVP group with was compared with the low expression circMVP group. Two-tailed paired *t*-test was used to analyze the intensity of METTL3 in cancer cells. **(C and D)** Univariate (C) and multivariate (D) Cox regression analysis was used to evaluate the association between circMVP, METTL3, B7-H3 and tumor-node-metastasis (TNM) stage. **(E)** Kaplan-Meier survival plots for the correlation between the expression of circMVP, high= 94 and low = 68 with the overall survival (OS) in patients. *p* < 0.05 in log rank tests. **(F)** Pearson correlation analysis of METTL3 (*p* = 0.0258*, r = 0.176, n = 172), B7-H3 (*p* = 0.0048*, r = 0.220, n = 172) and circMVP protein expression based on IHC staining scores. **(G)** Kaplan-Meier survival curves generated according to the expression of B7-H3 (CD276) in TCGA-COAD and TCGA-READ database (https://xenabrowser.net/datapages/). **(H)** Working model of the mechanism used by circMVP to facilitate the activation of METTL3-mediated RNA m6A modification which inhibits B7-H3 dependent anti-tumor immunity in CRC. Results are presented as mean±SD. *p < 0.05; **p < 0.01; ***p < 0.001 (Student's *t*-test; log-rank test). ISH, *in situ* hybridization; IHC, immunohistochemistry.

The correlation between circMVP and its downstream targets was examined by evaluating the expression of circMVP using ISH and the protein expression of β-catenin and B7-H3 in CRC tissues using IHC (Fig. 8A and Fig. S6D). The findings revealed that within the high circMVP expression group, 58.02% (94/162) of CRC tissue samples showed increased levels of β-catenin and B7-H3 expression. This figure notably surpassed those observed within the group characterized by low circMVP expression (Fig. 8B and Table S1). Univariate and multivariate Cox proportional hazards regression analyses identified circMVP, METTL3, and B7-H3 as significant prognostic factors for CRC (Fig. 8C and 8D). Survival analysis revealed that circMVP expression was associated with poor survival (*P* = 0.0009, Fig. 8E), and high expression of METTL3 (*P* = 0.0054) and B7-H3 (*P* = 0.0184) also suggested a poor prognosis in TCGA CRC patients (Fig. S6E and Fig. S6F). Additionally, the expression of both METTL3 and B7-H3 proteins in CRC samples was positively correlated with circMVP expression (Fig. 8F).

TCGA (COAD and READ) external CRC cohort was used to verify the role of B7-H3 as a potential diagnostic and therapeutic target for CRC. TCGA-COAD analysis showed that 50.00% (135/270) of CRC patients had high B7-H3 (CD276) expression and a poor prognosis (*P* = 0.046, Fig. 8G). TCGA-READ analysis showed that 11.96% (11/92) of CRC patients had high B7-H3 (CD276) expression and a poor prognosis (*P* = 0.020, Fig. 8G). Collectively, our results proved that METTL3 facilitated activation by circMVP-mediated m6A modification in CTNNB1 mRNA, and CTNNB1-translated protein β-catenin was upregulated in CRC which inhibited B7-H3 dependent immunosuppression in the TME.

## Discussion

CircRNAs have recently emerged as a large class of non-coding RNA that play critical roles in cancer development and progression through several epigenetic regulatory mechanisms of action. Accumulating evidence has shown that circRNAs also regulate the functions of proteins that regulate cancer^27^. The prominent physiological roles of circRNAs include acting as miRNA sponges, modulating transcriptional processes, and collaborating with RNA-binding proteins to exert pivotal biological functions^28^. This study demonstrated that circMVP directly binds to METTL3 protein and promotes stable protein translation of downstream mRNA through METTL3-mediated mRNA m6A methylation, thus promoting CRC growth and immunosuppression (Fig. 8H). Recent studies have found that circRNAs are involved in tumor regulation, and the roles of circRNAs in cancer are increasingly well understood, but their role in cancer immunotherapy remains unclear, particularly whether these circRNAs can serve as biomarkers to predict patient response to ICB treatment^29^. The present study systematically demonstrated the high circMVP expression in CRC and its specific binding to METTL3. Importantly, circMVP promoted METT3-mediated m6A modification of CTTNB1 mRNA and increased the translation efficiency of β-catenin protein. The circMVP/METT3 regulatory axis activates β-catenin signaling, a key molecule in transcriptional activation that promotes tumor progression.

METTL3 constitutes an integral part of the N6-methyltransferase complex implicated in numerous malignancies^30^. Our findings reveal the profound oncogenic significance of METTL3 in tumor advancement, although some studies have reported conflicting findings. Two previous independent investigations posited that METTL3 exerts an oncogenic influence on CRC through distinct downstream targets^31^,^32^,^33^. Indeed, in this work, circMVP was overexpressed in CRC cells and is mainly localized in the nucleus. circMVP improved the efficiency with which CTNNB1 mRNA is "Writer" labeled m6A by stabilizing METTL3 in the nucleus. In this study, we explored the molecular mechanism of how circMVP mediates the m6A modification of CTNNB1 gene by METTL3, and verified this through a series of *in vivo* and *in vitro* experiments. It was found that circMVP activated β-catenin signaling by interacting with METTL3 and affecting its m6A modification activity on CTNNB1.

Upon stimulation by Wnt ligands, β-catenin steadily accumulates and binds to TCF/LEF transcription factors, thereby initiating transcription^34^. The strong correlation between elevated levels of nuclear β-catenin and the progression of human colorectal carcinogenesis has been thoroughly established, leading to diminished patient survival^35^. Hence, targeting nuclear β-catenin is a promising strategy to combat cancer. Furthermore, particular residues in β-catenin that are crucial for its translocation into the nucleus have been identified. β-catenin binds to CD276 chromatin and increases CD276 transcript levels, which in turn upregulates B7-H3 expression, suggesting that circMVP/METTL3 promotes β-catenin regulation of B7-H3.

Recent therapeutic measures, such as immunotherapy involving immune-checkpoint inhibitors targeting PD1/PDL1, have exhibited only modest advancements in augmenting antineoplastic immune reactions in these individuals^36^. In preclinical assessments pertaining to cardiac allograft rejection, encephalomyelitis, and inflammation. B7-H3 has been found to be widely expressed in tumor cytoplasm and cell membranes, and has been shown to be involved in inflammatory responses, chemokine production, and complex tumor biological regulation in the cytoplasm^37^,^38^. *In vitro and in vivo* models have also demonstrated the function of B7-H3 as a T cell co-stimulatory protein, which is involved in specific immune regulation^6^,^8^. However, in cancer models, B7-H3 inhibits anti-tumor immunity by diminishing the cytotoxic activity of CD8+ T cells and NK cells, while independently facilitating migration, invasion, and metastasis unrelated to the immune system. Immune checkpoint inhibitors of B7-H3 may be a blocking therapy and may be an alternative supplementary therapy for anti-PD1/PDL1 immunotherapy^39^. This study demonstrated that circMVP promoted tumor formation in MC38 cells and enhanced the expression of B7-H3 in tumor cells. Analysis of infiltrated immune cells revealed that circMVP promoted tumor immune evasion through B7-H3. Interestingly, circMVP proposed in this study bound to METTL3 and stabilized the METTL3 protein, which also acted on circMVP to promote its nucleic acid chain stability. This also provides a new idea for the design of a vaccine combining immune checkpoint inhibitors and circRNA. Inhibition of circMVP combined with B7-H3 blockade effectively inhibited tumor growth and prolonged survival, achieving a better immunotherapeutic effect.

## Conclusion

This study demonstrates a new regulatory function of circMVP in CRC that facilitates cancer cell growth and immunosuppression. circMVP binds to METTL3 to regulate m6A methylation of CTNNB1 mRNA, upregulates β-catenin protein, and activates B7-H3 expression. Inhibition of circMVP expression disturbed B7-H3 dependent anti-tumor immunity in CRC. The identification of the circMVP/METTL3/β-catenin/B7-H3 signaling axis provides new insights into the molecular mechanisms underlying immunosuppression and tumor progression, identifying new immunotherapeutic targets for CRC.

## Supplementary Material

Supplementary figures and tables.

## Abbreviations

circRNA: Circular RNA
m6A: N6-methyladenosine
CRC: Colorectal cancer
ICB: Immune checkpoint blockade
TME: Tumor microenvironment
FISH: *in situ* hybridization
RIP: RNA immunocoprecipitation
scRNA: Single-cell RNA sequencing
MeRIP: Methylated RNA immunocoprecipitation
ChIP: Chromatin immunoprecipitation
MVP: Major vault protein
METTL3: Methyltransferase 3
CTNNB1: catenin beta 1
B7-H3: B7 family homolog 3
UMAP: Uniform manifold approximation and projection
EMSA: Electrophoretic mobility shift assay
