# Supplementary material for: CircMVP promotes METTL3 activation mediated CTNNB1 m6A modification in the inhibition of colorectal cancer in B7-H3 dependence antitumor immunity
Source: Int J Biol Sci. 2025 Jan 1;21(1):306–27. doi: 10.7150/ijbs.105324 (PMC11667818; doi:10.7150/ijbs.105324)
Supplement: Supplementary file 1 — Supplementary figures and tables. [file ijbsv21p0306s1.pdf]

Figure S1. circMVP expression in CRC

(A) Heatmap plot of differentially expressed circRNAs from transcriptome analysis of colorectal cancer tissues (3) and paired adjacent normal tissues. **(B)** Information and molecular predictions of CircMVP (hsa_circ_0000688) included in CSCD (Cancer-Specific CircRNA Database). **(C)** Combining PCR with an electrophoresis assay indicated the presence of circMVP using divergent and convergent primers from cDNA or genomic DNA (gDNA) in DLD1, HCT8 and HCT116 cells. **(D)** The expression level of circMVP in CRC cell line (DLD1, HCT8, HCT116, RKO) and 293T were detected by separation of nucleus and cytoplasm and qRT-PCR. **(E)** Expression of circMVP was examined by ISH in normal epithelial tissues (n=46) and CRC tissues (n=46), (Student's t-test; log-rank test). **(F)** Features plot showed distribution and expression levels of circMVP and its hostgene: MVP. **(G)** CircMVP knockdown and overexpression cell models were constructed in DLD-1 and HCT8 cells, respectively, and transfection effect was verified by qRT-PCR. Data were presented as mean±SD. *p<0.05; **p<0.01; ***p<0.001(Student's t-test; log-rank test). CRC, colorectal cancer; ISH, *in situ* hybridization.

Figure S2. CircMVP facilitates CRC growth

(A) The effects of circMVP on cell proliferation in HCT116 and RKO. **(B and C)** Transwell-matrigel was used to detect the effect of circMVP on the invasion ability of cancer cells. Scale bars, 300 µm. **(D and E)** Transwell was used to detect the effect of circMVP on the migration ability of cancer cells. Scale bars, 300 µm. **(F)** The expression of circMVP in mouse cell lines (B16, BMDM, MC38, 4T1 and CT26) was detected by PCR and analyzed by gel electrophoresis. **(G)** The effects of circMVP on cell proliferation in MC38. **(H and I)** Transwell-matrigel and transwell were used to detect the effect of circMVP on the invasion and migration ability of MC38. Scale bars, 300 µm. **(J)** Pathological section and Spatial transcriptome MVP in the CRC samples from PRJNA942633. Each dot was a cell colored by its analyzed cell types. Data are presented as mean±SD. *p<0.05; **p<0.01; ***p<0.001(Student's t-test; log-rank test). CRC, colorectal cancer.

Figure S3. CircMVP interacts with METTL3

(A) The proteins enriched by circMVP RNA pull-down were obtained with 40kDa-72kDa specific

differential expression bands, and the METTL3 protein was found by MS analysis, peak map and proteins abundance table were displayed. **(B)** RNA-EMSA was used to detect the binding between circMVP and METTL3 protein. **(C)** CircMVP expression inhibited the ubiquitin modification of METTL1. HCT8 cells were transfected with circMVP or METTL3 and treated with MG132 (20 mmol/mL) for 3 hours, the binding ubiquntin level was detected. **(D, E, F and G)** The combination of METTL3 and circMVP was detected by RIP, and the fluorescence signal of RNA was obtained by PCR amplification and then gel electrophoresis to identify the specificity of circMVP, respectively in DLD1, 293T, HCT8 and MC38. **(H)** The protein levels of METTL1 were measured in si-circMVP DLD1 cells by Western blot. Cells were treated with CHX (50 μg/mL) for 3 hours. MS, Mass spectrum.

Figure S4. CircMVP/METTL3/β-catenin regulates B7-H3 expression

(A) Transcriptome analysis differentially expressed genes in HCT8-circMVP compared with HCT8-NC in scatter plot. **(B)** KEGG demonstrated the β-catenin signaling pathway (https://www.kegg.jp/pathway/map04310). **(C)** GSEA plot demonstrated the Wnt/β-catenin signaling pathway in HCT8-circMVP and HCT8-NC. **(D and E)** SRAMP predicted the sites and confidence intervals of Homo sapiens (CTNNB1) and mouse (Ctnnb1) mRNA modified by m6A, respectively (http://www.cuilab.cn/sramp). **(F)** Transcriptome of RNAseq analysis of basic expression levels of Ctnnb1 in mouse cell lines (GEO: GSE18398) and expression of β-catenin protein in mouse cell lines (B16, BMDM, MC38, 4T1 and CT26) was detected by PCR. **(G and H)** Transcriptome and qRT-PCT were used to analyze the effect of circMVP on downstream regulatory gene expression of β-catenin signaling pathway. **(I)** The effect of circMVP/METTL3 regulation on CTNNB1 transcription level was detected by qRT-PCR. KEGG, Kyoto Encyclopedia of Genes and Genomes; GSEA, Gene Set EnrichmentAnalysis; SRAMP,sequence-based RNA adenosine methylation site predictor.

Figure S5. Single cell sequencing of CircMVP MC38 mouse tumor model

(A) UMAP plot showed the clusters of scRNA-seq in tumor tissue of MC38 cells (NC vs. circMVP). Each dot is a cell colored by its analyzed cell types. **(B)** Feature plots showed tumor Epithelial cell signatures (Grb2 and Krt14) transcript levels in MC38-NC and MC38-circMVP. **(C**

and D) Violin plots showed tumor Immune checkpoint signatures ranscript levels in MC38-NC and MC38-circMVP. **(E)** GO pathway enrichment terms of the differentially expressed genes in CD8+ T cells of MC38-circMVP compared with CD8+ T cells of MC38-NC. **(F)** scRNA sequencing analysis. The expression levels of NK cells function markers Klrd1, Gzmb and Tigit infiltrated by MC38-circMVP were compared with those of MC38-NC. **(G)** The expression levels of CD276 in MC38-circMVP was compared with those of MC38-NC. **(H)** scRNA sequencing analysis. The expression levels of Mettl3 and Tthdf1 by MC38-circMVP were compared with those of MC38-NC. UMAP, Uniform Manifold Approximation and Projection; TAM,Tumor-associated macrophages; GO, Gene ontology.

Figure S6. β-catenin expression, METTL3 and B7-H3 survival analysis

(A) qRT-PCT were used to analyze the effect of circMVP on downstream regulatory gene expression of CD276; ChIP-seq analysis of β-catenin acts on heat maps of CD276 chromatin Peak distribution upstream and downstream of TSS. **(B)** Expression of circMVP in in vivo tumorigenic tissue detected by ISH. **(C)** IGV tracks showing m6A peaks distribution on CD276 transcript from MeRIP-seq data in PRJNA927821. **(D)** Expression of β-catenin proteins detected by IHC in CRC tissue. Scale bar, 60 μm. **(E)** Kaplan-Meier survival plots for the correlation between the expression of METTL3, high= 50 and low = 112 with the overall survival (OS) in patients. **(F)** Kaplan-Meier survival plots for the correlation between the expression of B7-H3, high= 82 and low = 80 with the overall survival (OS) in patients. ChIP, Chromatin immunoprecipitation; TSS, Transcription start site.

Figure S1

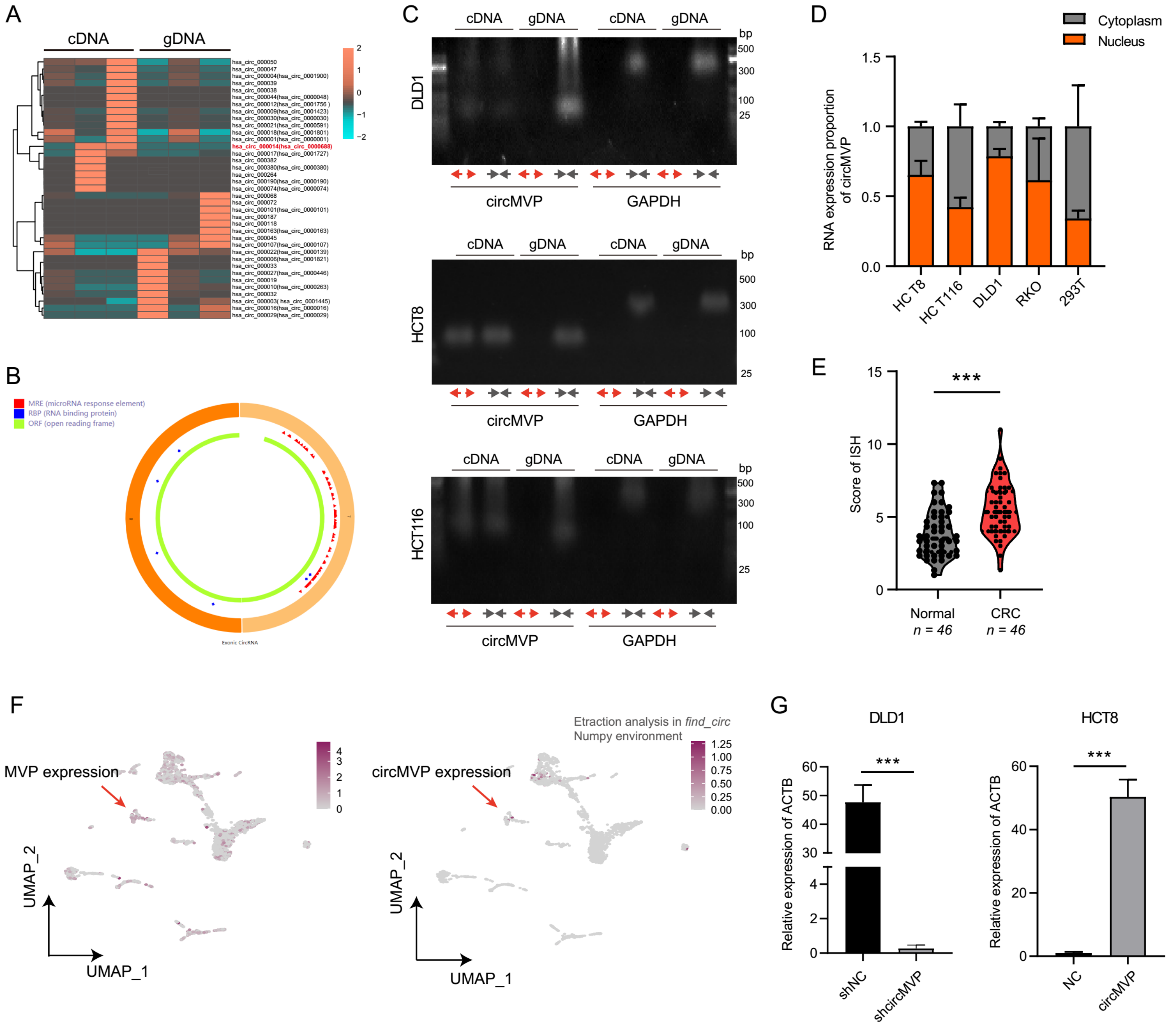

A
cDNA
gDNA
hsa_circ_000014(hsa_circ_0000688)
B
MRE (microRNA response element)
RBP (RNA binding protein)
ORF (open reading frame)
Exonic CircRNA
C
cDNA
gDNA
bp
500
300
100
25
DLD1
HCT8
HCT116
circMVP
GAPDH
D
Cytoplasm
Nucleus
RNA expression proportion of circMVP
1.5
1.0
0.5
0.0
HCT8
HCT116
DLD1
RKO
293T
E
Score of ISH
15
10
5
0
***
Normal
n = 46
CRC
n = 46
F
MVP expression
circMVP expression
Etraction analysis in find_circ
Numpy environment
UMAP_1
UMAP_2
G
DLD1
HCT8
Relative expression of ACTB
60
50
40
30
4
2
0
20
***
shNC
shcircMVP
NC
circMVP

Figure S2

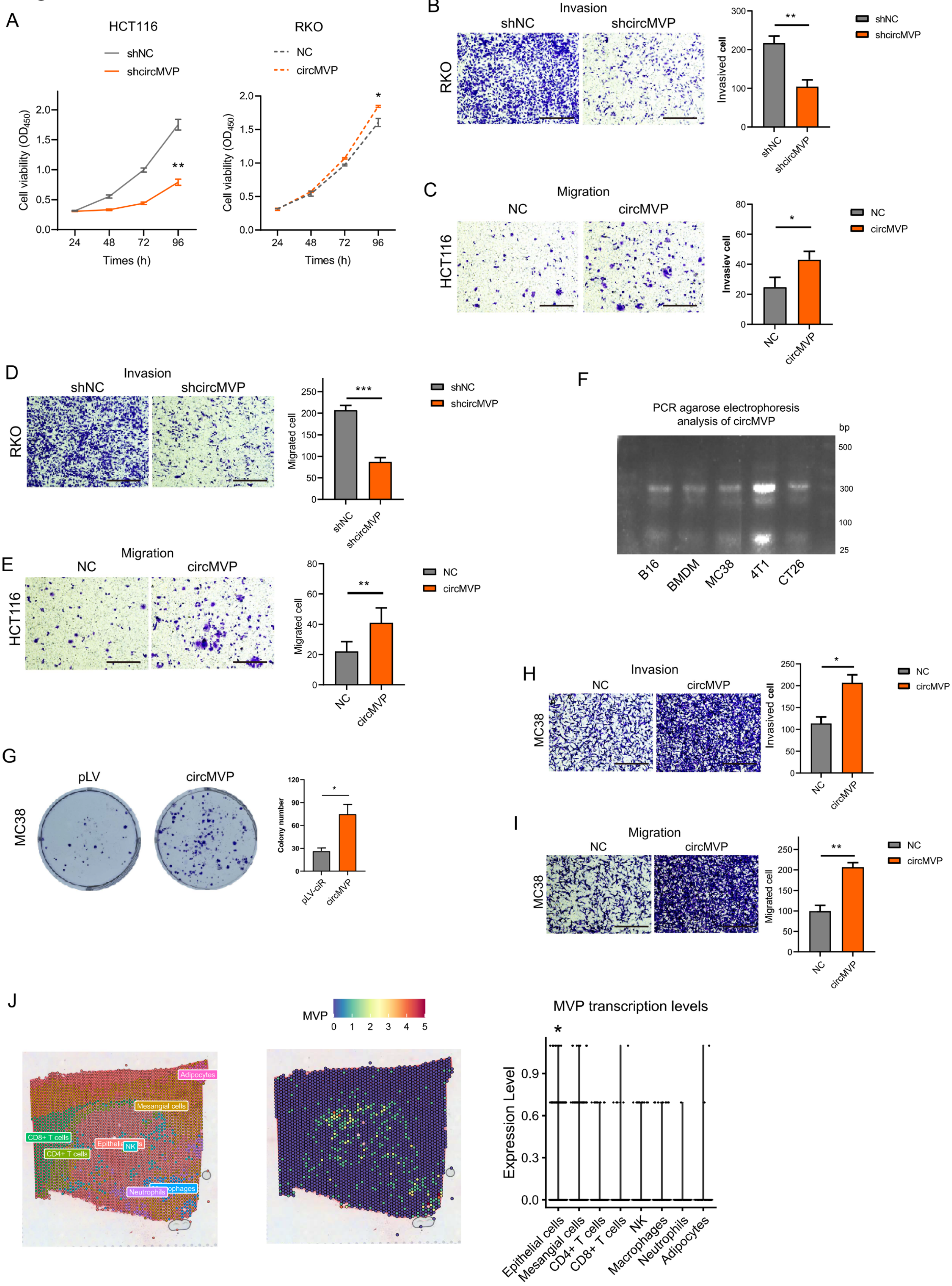
A
HCT116
shNC
shcircMVP
Cell viability (OD450)
2.0
1.5
1.0
0.5
0.0
24
48
72
96
**
Times (h)
RKO
NC
circMVP
*
B
Invasion
shNC
shcircMVP
RKO
Invasived cell
300
200
100
0
C
Migration
NC
circMVP
HCT116
Invasiev cell
80
60
40
20
D
Invasion
Migrated cell
250
150
50
***
E
Migration
F
PCR agarose electrophoresis
analysis of circMVP
bp
500
300
100
25
B16
BMDM
MC38
4T1
CT26
G
pLV
circMVP
MC38
Colony number
120
90
30
pLV-ciR
H
Invasion
I
Migration
J
MVP
0
1
2
3
4
5
Adipocytes
Mesangial cells
CD8+ T cells
CD4+ T cells
Neutrophils
MVP transcription levels
Expression Level
0.9
0.6
0.3
0.0
Epithelial cells
Mesangial cells
CD4+ T cells
CD8+ T cells
NK
Macrophages
Neutrophils
Adipocytes

Figure S3

A

Proteins detected by circMVP Pull-down
Mass spectrometry

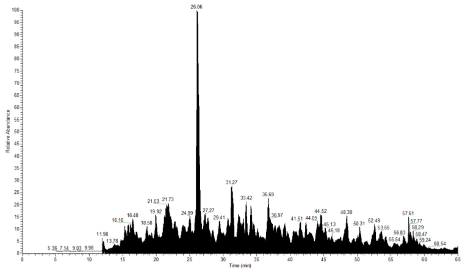

TOP15 proteins in MS

Protein FDR	Accession	Sum PEP Score	AAs	Molecular Function	Abundances
High	P35908	281.966	639	bone, tooth or skin structural activity;cytoskeletal activity	2571029694
High	P13645	279.866	584	bone, tooth or skin structural activity;other molecular function	4546927467
High	P68104	259.243	462	nucleic acid binding activity;other molecular function	9089267112
High	Q9UHB6	252.745	759	cytoskeletal activity;other molecular function	4591731775
High	P04264	247.527	644	bone, tooth or skin structural activity;other molecular function	6122395367
High	Q9Y5B9	235.682	1047	other molecular function	983139816.6
High	P19338	234.339	710	nucleic acid binding activity;other molecular function	15911389082
High	Q86U44	218.733	580	mRNA (2'-O-methyladenosine-N6-)-methyltransferase activity; mRNA binding;other molecular function	356360533.1
High	P09429	213.081	215	signal transduction activity or receptor binding;nucleic acid binding activity;other molecular function	8005659883
High	P13639	209.514	858	translation activity;nucleic acid binding activity;other molecular function	812448307.4
High	Q9BUF5	205.62	446	cytoskeletal activity;other molecular function	101169641.3
High	P07355	197.655	339	transporter activity;enzyme regulator activity;cytoskeletal activity;other molecular function	1885017542
High	P35527	193.723	623	cytoskeletal activity	1475197347
High	P14618	191.383	531	nucleic acid binding activity;kinase activity;other molecular function	726779818.3
High	P02545	179.879	664	cytoskeletal activity;other molecular function	792568860.1

METTL3

B

RNA-EMSA

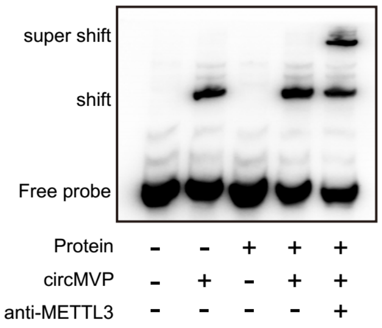

C

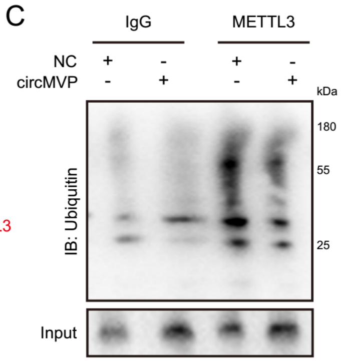

D

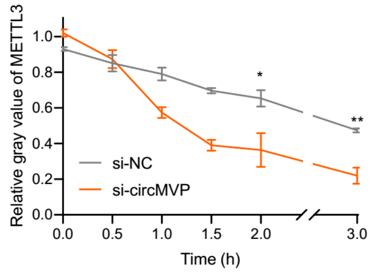

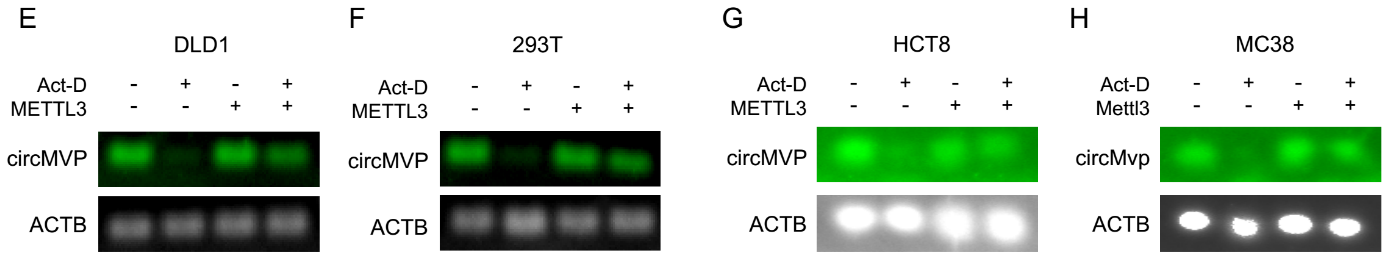

Figure S4

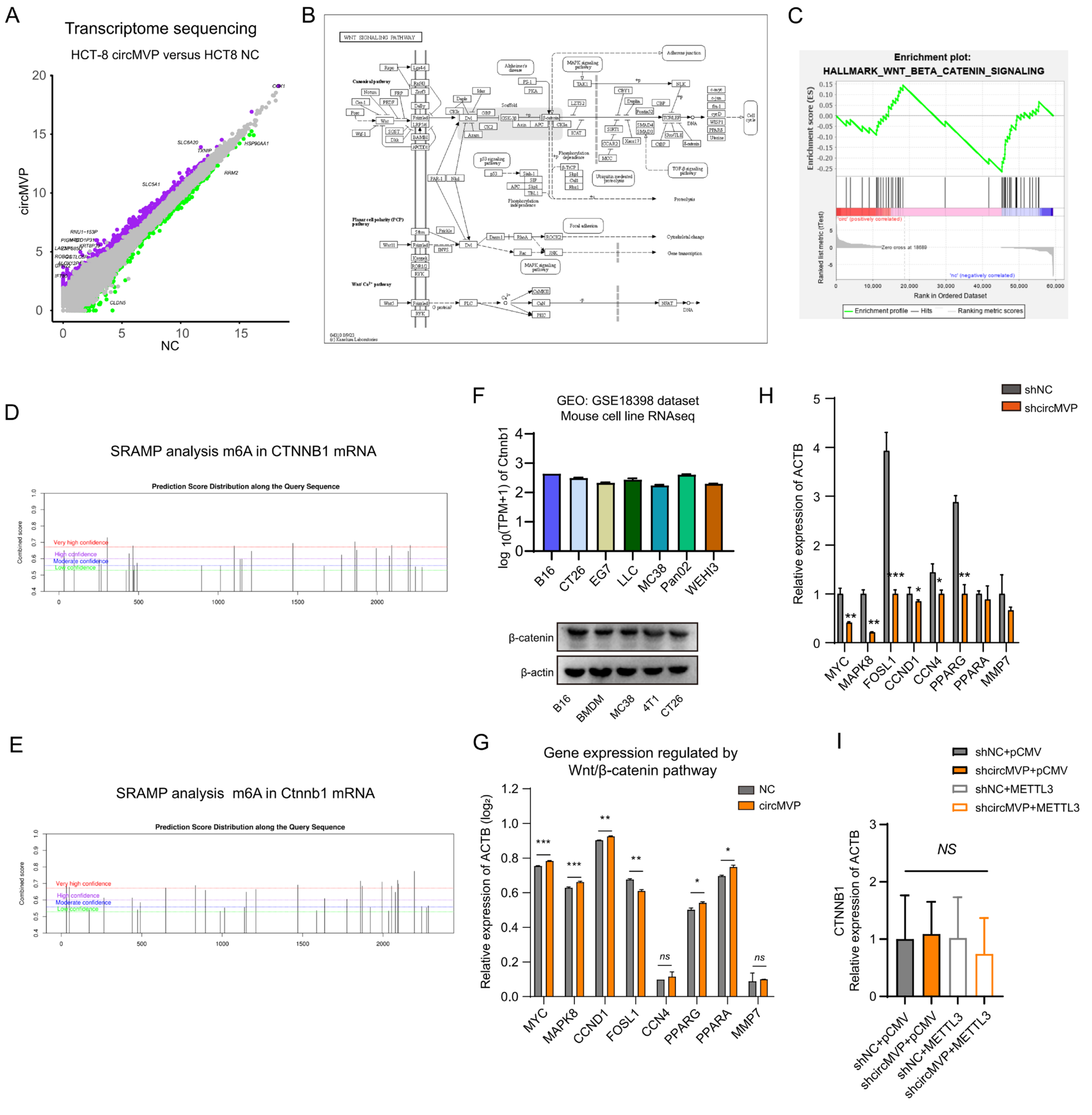
A
Transcriptome sequencing
HCT-8 circMVP versus HCT8 NC
circMVP
NC
B
WNT SIGNALING PATHWAY
C
Enrichment plot:
HALLMARK_WNT_BETA_CATENIN_SIGNALING
Enrichment score (ES)
Ranked list metric (tTest)
Rank in Ordered Dataset
Enrichment profile
Hits
Ranking metric scores
D
SRAMP analysis m6A in CTNNB1 mRNA
Prediction Score Distribution along the Query Sequence
Very high confidence
High confidence
Moderate confidence
Low confidence
Combined score
E
SRAMP analysis m6A in Ctnnb1 mRNA
Prediction Score Distribution along the Query Sequence
F
GEO: GSE18398 dataset
Mouse cell line RNAseq
log 10(TPM+1) of Ctnnb1
B16
CT26
EG7
LLC
MC38
Pan02
WEHI3
β-catenin
β-actin
B16
BMDM
MC38
4T1
CT26
G
Gene expression regulated by
Wnt/β-catenin pathway
Relative expression of ACTB (log2)
NC
circMVP
MYC
MAPK8
CCND1
FOSL1
CCN4
PPARG
PPARA
MMP7
H
shNC
shcircMVP
Relative expression of ACTB
MYC
MAPK8
FOSL1
CCND1
CCN4
PPARG
PPARA
MMP7
I
shNC+pCMV
shcircMVP+pCMV
shNC+METTL3
shcircMVP+METTL3
CTNNB1
Relative expression of ACTB
NS

Figure S5

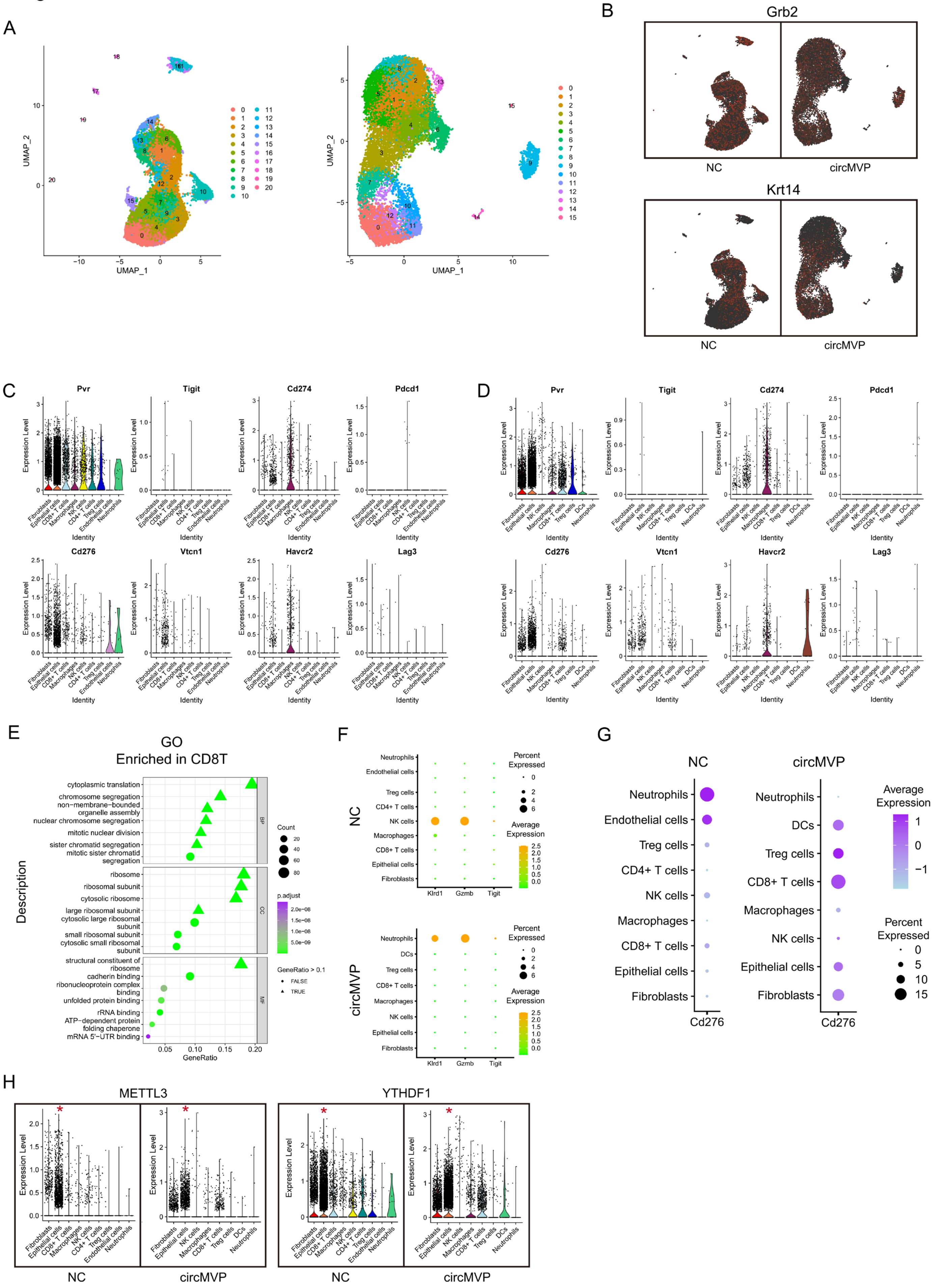
A
UMAP_1
UMAP_2
B
Grb2
NC
circMVP
Krt14
NC
circMVP
C
D
Pvr
Tigit
Cd274
Pdcd1
Cd276
Vtcn1
Havcr2
Lag3
Expression Level
Identity
Fibroblasts
Epithelial cells
CD8+ T cells
Macrophages
NK cells
CD4+ T cells
Treg cells
Endothelial cells
Neutrophils
DCs
E
GO
Enriched in CD8T
Description
cytoplasmic translation
chromosome segregation
non−membrane−bounded organelle assembly
nuclear chromosome segregation
mitotic nuclear division
sister chromatid segregation
mitotic sister chromatid segregation
ribosome
ribosomal subunit
cytosolic ribosome
large ribosomal subunit
cytosolic large ribosomal subunit
small ribosomal subunit
cytosolic small ribosomal subunit
structural constituent of ribosome
cadherin binding
ribonucleoprotein complex binding
unfolded protein binding
rRNA binding
ATP−dependent protein folding chaperone
mRNA 5'−UTR binding
BP
CC
MF
Count
p.adjust
GeneRatio > 0.1
FALSE
TRUE
GeneRatio
F
NC
circMVP
Percent Expressed
Average Expression
Klrd1
Gzmb
Tigit
G
NC
circMVP
Average Expression
Percent Expressed
Cd276
H
METTL3
YTHDF1
NC
circMVP
NC
circMVP

Figure S6

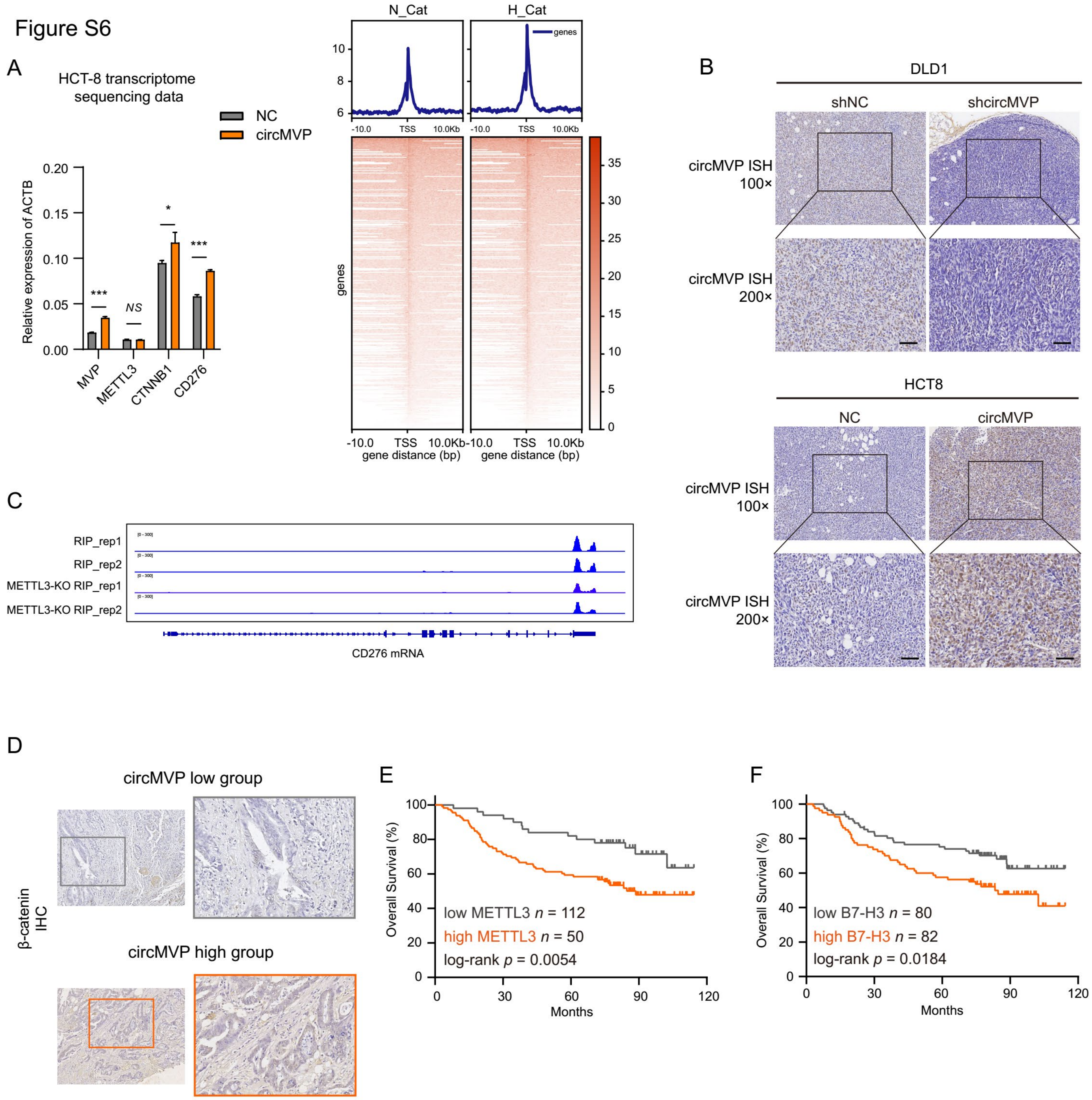
A
HCT-8 transcriptome sequencing data
NC
circMVP
Relative expression of ACTB
0.20
0.15
0.10
0.05
0.00
***
NS
*
***
MVP
METTL3
CTNNB1
CD276
N_Cat
H_Cat
genes
-10.0
TSS
10.0Kb
gene distance (bp)
B
DLD1
shNC
shcircMVP
circMVP ISH 100×
circMVP ISH 200×
HCT8
NC
circMVP
C
RIP_rep1
RIP_rep2
METTL3-KO RIP_rep1
METTL3-KO RIP_rep2
CD276 mRNA
D
circMVP low group
circMVP high group
β-catenin IHC
E
Overall Survival (%)
low METTL3 n = 112
high METTL3 n = 50
log-rank p = 0.0054
Months
F
low B7-H3 n = 80
high B7-H3 n = 82
log-rank p = 0.0184

Table S1. Colorectal cancer pathological characteristics table of circMVP in Cohort of affiliated hospital of Jiangnan University.

Clinical parameters	ISH of circMVP		$\chi 2$	*P value*
	Low (n=92)	High (n=70)		
Gender			0.096	0.756
Female	43(46.7%)	31(44.3%)		
Male	49(53.3%)	39(55.7%)		
Age			2.127	0.145
＜60	42(45.7%)	24(34.3%)		
≥60	50(54.3%)	46(65.7%)		
Location			5.535	**0.019***
Colon	28(30.4%)	34(48.6%)		
Rectum	64(69.6%)	36(51.4%)		
T stage			4.017	**0.045***
T1-T2	30(32.6%)	13(18.6%)		
T3-T4	62(67.4%)	57(81.4%)		
N stage			7.685	**0.006****
N0	57(62.0%)	28(40.0%)		
N1-N4	35(38.0%)	42(60.0%)		
M stage			13.285	**<0.001*****
M0	89(96.7%)	55(78.6%)		
M1	3(3.3%)	15(21.4%)		
METTL3			3.704	0.054
IHC score≤3	34(37.0%)	16(22.9%)		
IHC score＞3	58(63.0%)	54(77.1%)		
B7-H3			8.953	**0.004****
IHC score≤4	56(60.9%)	26(37.1%)		
IHC score＞4	36(39.1%)	44(62.9%)		

Pearson χ2 test or Fisher exact test. *$P < 0.05$, **$P < 0.01$, ***$P < 0.001$.

Table S2. Primer and sequences.

**Primers for PCR and real time PCR**		
**Primers**	Sequences-F	Sequences-R
circMVP#1	5′-CCCGAAAGATCCATGGAGAGA-3′	5′-GGCTGGGTGGTGAATGTAGA-3′
circMVP#2	5′-AAGTGGAGGTCGTGGAGATC-3′	5′-CCACCACCTTGTCTCCATCT-3′
circMVP-convergent	5′-CTGTCACCCTCTCCTTGCC-3′	5′-ATGAGTGGCTTTTCGAGGGA-3′
MVP	5′-ATCGAAACGGCGGATCATGCCA-3′	5′-GGCATCACCTACAAAGTCTGGC-3′
β-actin (ACTB) for human	5′-AACTCCATCATGAAGTGTGACG-3′	5′-GATCCACATCTGCTGGAAGG-3′
β-actin (ACTB) for mouse	5′-CATTGCTGACAGGATGCAGAAGG-3′	5′-TGCTGGAAGGTGGACAGTGAGG-3′
GAPDH for human	5′-GTCTCCTCTGACTTCAACAGCG-3′	5′-ACCACCCTGTTGCTGTAGCCAA-3′
Gapdh for mouse	5′-CATCACTGCCACCCAGAAGACTG-3′	5′-ATGCCAGTGAGCTTCCCGTTCAG-3′
U6	5′-CTCGCTTCGGCAGCACA-3′	5′-AACGCTTCACGAATTTGCGT-3′
CTNNB1 for human	5′-CACAAGCAGAGTGCTGAAGGTG-3′	5′-GATTCCTGAGAGTCCAAAGACAG-3′
Ctnnb1 for mouse	5′-GTTCGCCTTCATTATGGACTGCC-3′	5′-ATAGCACCCTGTTCCCGCAAAG-3′
CD276 for human	5′-CTGGCTTTCGTGTGCTGGAGAA-3′	5′-GCTGTCAGAGTGTTTCAGAGGC-3′
Cd276 for mouse	5′-AGCATCCAGGACTTTGACAGCG-3′	5′-CGTGATGGTCACCATGTTCCCT-3′

CD276 for ChIP	5′-CTGGGAGCACTGTGGTTCT-3′	5′-TTGGTATCTGTCAGCTGCCA-3′
METTL3 for human	5′-CTATCTCCTGGCACTCGCAAGA-3′	5′-GCTTGAACCGTGCAACCACATC-3′
Mettl3 for mouse	5′-CAGTGCTACAGGATGACGGCTT-3′	5′-CCGTCCTAATGATGCGCTGCAG-3′
CTNNB1 for RIP	5′-CTCCTAGTGTTTGGCCTGGA-3′	5′-CGGTTCCATGCATACACAGG-3′
MYC	5′-CCTGGTGCTCCATGAGGAGAC-3′	5′-CAGACTCTGACCTTTTGCCAGG-3′
MAPK8	5′-GACGCCTTATGTAGTGACTCGC-3′	5′-TCCTGGAAAGAGGATTTTGTGGC-3′
FOSL1	5′-GGAGGAAGGAACTGACCGACTT-3′	5′-CTCTAGGCGCTCCTTCTGCTTC-3′
CCND1	5′-TCTACACCGACAACTCCATCCG-3′	5′-TCTGGCATTTTGGAGAGGAAGTG-3′
CCN4	5′-AAGAGAGCCGCCTCTGCAACTT-3′	5′-TCATGGATGCCTCTGGCTGGTA-3′
PPARG	5′-AGCCTGCGAAAGCCTTTTGGTG-3′	5′-GGCTTCACATTCAGCAAACCTGG-3′
PPARA	5′-TCGGCGAGGATAGTTCTGGAAG-3′	5′-GACCACAGGATAAGTCACCGAG-3′
MMP7	5′-TCGGAGGAGATGCTCACTTCGA-3′	5′-GGATCAGAGGAATGTCCCATACC-3′
**Sequences for gene knockdown**		
Si-circMVP #1	GGAUAAAGAUGGAGACAAG	CUUGUCUCCAUCUUUAUCC
Si-circMVP #2	GGAGAGGGUGACAGGACAU	AUGUCCUGUCACCCUCUCC
sh-circMVP #1	GGATAAAGATGGAGACAAG	CTTGTCTCCATCTTTATCC
sh-circMVP	GGAGAGGGTGACAGGACAT	ATGTCCTGTCACCCTCTCC

#2		
Si-METTL3	CAGUGGAUCUGUUGUGAUAUC	UAUCACAACAGAUCCACUGAG
**Gene sequences**		
circMVP	GACATCACACCCCTGCAGGTGGTTCTGCCCAACACTGCCCTCCATCTAAAGGCGCTGCTTGATTTTGAGGATAAAGATGGAGACAAGGTGGTGGCAGGAGATGAGTGGCTTTTCGAGGGACCTGGCACGTACATCCCCCGGAAGGAAGTGGAGGTCGTGGAGATCATTCAGGCCACCATCATCAGGCAGAACCAGGCTCTGCGGCTCAGGGCCCGCAAGGAGTGCTGGGACCGGGACGGCAAGGAGAGGGTGACAG	
circMVP Probe	5’-Cy3/Biotin-TGTGATGTCCTGTCACCCTCT-3’	

Table S3. The detailed antibody information used in this study.

Antibodies	Source	Cat#
Anti-β-actin	Cell Signaling Technology	Cat#3700
Anti-METTL3 (Rabbit)	Cell Signaling Technology	Cat#86132
Anti-METTL3 (Mouse)	Proteintech	Cat#67733-1-Ig
Anti-YTHDF1	Cell Signaling Technology	Cat#57530
Anti-B7-H3	Cell Signaling Technology	Cat#14058
Anti-β-catenin (Rabbit)	Cell Signaling Technology	Cat#8480
Anti-β-catenin (Mouse)	Proteintech	Cat#66379-1-Ig
Anti-m6A	Cell Signaling Technology	Cat#56593
Anti-Histone 3	Cell Signaling Technology	Cat#4499
Methylene blue	Sigma-Aldrich	Cat#M9140
Phalloidin	Beyotime	Cat#25682-1-AP
Omburtama	Selleck	Cat#1895083-75-6
Mouse IgG1	Selleck	Cat#MOPC-21
Anti-Mouse CD16/CD32, APC	MultiScience	Cat#F210163203
FoxP3/Transcription Factor Staining Buffer Kit	MultiScience	Cat#IC001
Anti-CD326 (EpCAM) Monoclonal, PE, eBioscience™	Invitrogen	Cat#12-9326-42
Anti-Mouse CD45, FITC	MultiScience	Cat#F2104501
Anti-Mouse CD3ε, PE	MultiScience	Cat#F2100302
Anti-Mouse CD4, APC	MultiScience	Cat#F2100403
Anti-Mouse CD8α, FITC	MultiScience	Cat#F2100801
Anti-Mouse NK1.1(CD161)，PE-Cy7	MultiScience	Cat#F2116105
Anti-Human/Mouse CD11b, PerCP-Cy5.5	MultiScience	Cat#F41011b04
Anti-Mouse F4/80, APC	MultiScience	Cat#F21480A03
Anti-CD206/MRC1 Rabbit mAb (Alexa Fluor® 594)	MultiScience	Cat#59414

eBioscience™ Fixable Viability Dye	Invitrogen	Cat#65-2860-40
Anti-mouse-IgG-HRP for WB	Promega	Cat#W4021
Anti-rabbit-IgG-HRP for WB	Promega	Cat#W4011
Cy3-labeled Goat Anti-Mouse IgG (H+L)	Beyotime	Cat#A0521
Cy3-labeled Goat Anti-Rabbit IgG (H+L)	Beyotime	Cat#A0516
FITC-labeled Goat Anti-Mouse IgG (H+L)	Beyotime	Cat#A0568
FITC-labeled Goat Anti-Rabbit IgG (H+L)	Beyotime	Cat#A0562
Anti-mouse-IgG-HRP for IHC	Beyotime	Cat#A0216
Anti-rabbit-IgG-HRP for IHC	Beyotime	Cat#A0208
Multiplex Fluorescence IF Staining Kit	ABSIN	Cat#abs50013
DAB detection kit	ABSIN	Cat#abs9210
ChIP or CUT&RUN Assay Kit	Vazyme	Cat#HD101

Table S4. Differentially expressed circRNAs in Colorectal cancer.

circBASE ID	circBANK ID	logFC	P.Value	Regulation
**hsa_circ_000014**		1.271174896	0.038990425	UP
hsa_circ_000050		0.754344802	0.045015071	UP
hsa_circ_000017		1.123316537	0.089556881	UP
hsa_circ_000018		0.526715161	0.129154697	UP
hsa_circ_000047		0.74216951	0.137060076	UP
hsa_circ_000030	hsa_circ_0000030	1.135330787	0.209592288	UP
hsa_circ_000004		0.602451641	0.219659551	UP
hsa_circ_000021		0.744220252	0.222333938	UP
hsa_circ_000009		0.528320834	0.226712002	UP
hsa_circ_000380	hsa_circ_0000380	1.092661582	0.236663442	UP
hsa_circ_000044		0.754344802	0.241032831	UP
hsa_circ_000001	hsa_circ_0000001	0.62917509	0.242771623	UP
hsa_circ_000012		0.654491375	0.24374094	UP
hsa_circ_000039		0.356796443	0.244314542	UP

hsa_circ_000038		0.528320834	0.24951087	UP
hsa_circ_000264		0.459503874	0.254739529	UP
hsa_circ_000382		0.356796443	0.268399785	UP
hsa_circ_000190	hsa_circ_0000190	0.255178249	0.298624688	UP
hsa_circ_000074	hsa_circ_0000074	0.255178249	0.298624688	UP
hsa_circ_000022		-0.635630199	0.060452328	DOWN
hsa_circ_000016	hsa_circ_0000016	-0.868023775	0.061351332	DOWN
hsa_circ_000029	hsa_circ_0000029	-1.101016695	0.066824723	DOWN
hsa_circ_000010	hsa_circ_0000263	-0.816300317	0.087858824	DOWN
hsa_circ_000003		-0.489828428	0.095842268	DOWN
hsa_circ_000032		-1.137593842	0.099286803	DOWN
hsa_circ_000068		-0.836987306	0.21413584	DOWN
hsa_circ_000027		-0.615998969	0.218970453	DOWN
hsa_circ_000045		-0.512017633	0.232109455	DOWN
hsa_circ_000019		-0.40054462	0.237044391	DOWN
hsa_circ_000006	hsa_circ_0001821	-0.712501175	0.242032814	DOWN
hsa_circ_000033		-0.477653136	0.253149432	DOWN
hsa_circ_000072		-0.379167841	0.264530021	DOWN
hsa_circ_000107	hsa_circ_0000107	-0.421011469	0.266238623	DOWN
hsa_circ_000118		-0.308666473	0.27944487	DOWN
hsa_circ_000163	hsa_circ_0000163	-0.308666473	0.27944487	DOWN
hsa_circ_000187		-0.282665636	0.287588805	DOWN
hsa_circ_000101	hsa_circ_0000101	-0.282665636	0.287588805	DOWN

Table S5.TOP100 protein detected by circRNA Pull-down Mass spectrometry.

Protein FD	Accession	Description	Sum PEP S	Covera	# Peptic	# PSMs	# Uniq	# AAs	Biological	Cellular C	Molecular	Abundances
High	P35908	Keratin, type II cytoskeletal 2 epidermal OS=Homo sapiens OX=9606 GN=KRT2 PE=1 SV=2	281.966	67	38	91	29	639	cell cycle (	plasma me	bone, tootl	2571029694
High	P13645	Keratin, type I cytoskeletal 10 OS=Homo sapiens OX=9606 GN=KRT10 PE=1 SV=6	279.866	61	33	102	31	584	cell organi	plasma me	bone, tootl	4546927467
High	P68104	Elongation factor 1-alpha 1 OS=Homo sapiens OX=9606 GN=EEF1A1 PE=1 SV=1	259.243	54	20	139	7	462	protein me	non-structu	nucleic aci	9089267112
High	Q9UHB6	LIM domain and actin-binding protein 1 OS=Homo sapiens OX=9606 GN=LIMA1 PE=1 SV=1	252.745	60	38	76	38	759	cell organi	plasma me	cytoskeleta	4591731775
High	P04264	Keratin, type II cytoskeletal 1 OS=Homo sapiens OX=9606 GN=KRT1 PE=1 SV=6	247.527	48	39	122	32	644	cell organi	non-structu	bone, tootl	6122395367
High	Q9Y5B9	FACT complex subunit SPT16 OS=Homo sapiens OX=9606 GN=SUPT16H PE=1 SV=1	235.682	39	40	79	40	1047	cell organi	nucleus	other mole	983139817
High	P19338	Nucleolin OS=Homo sapiens OX=9606 GN=NCL PE=1 SV=3	234.339	44	38	251	38	710	developme	plasma me	nucleic aci	1.5911E+10
High	Q86U44	Methyltransferase-like protein 3 OS=Homo sapiens OX=9606 GN=METTL3 PE=1 SV=2	218.733	65	24	240	7	580	N6-adenos	cytosol; R	mRNA (2	356360533
High	P09429	High mobility group protein B1 OS=Homo sapiens OX=9606 GN=HMGB1 PE=1 SV=3	213.081	54	20	135	18	215	cell organi	non-structu	signal tran	8005659883
High	P13639	Elongation factor 2 OS=Homo sapiens OX=9606 GN=EEF2 PE=1 SV=4	209.514	40	35	65	34	858	protein me	non-structu	translation	812448307
High	Q9BUF5	Tubulin beta-6 chain OS=Homo sapiens OX=9606 GN=TUBB6 PE=1 SV=1	205.62	65	22	179	9	446	cell cycle (	cytoskeleto	cytoskeleta	101169641
High	P07355	Annexin A2 OS=Homo sapiens OX=9606 GN=ANXA2 PE=1 SV=2	197.655	65	28	71	28	339	cell organi	non-structu	transporter	1885017542
High	P35527	Keratin, type I cytoskeletal 9 OS=Homo sapiens OX=9606 GN=KRT9 PE=1 SV=3	193.723	53	27	61	27	623	cell organi	cytosol;cy	cytoskeleta	1475197347
High	P14618	Pyruvate kinase PKM OS=Homo sapiens OX=9606 GN=PKM PE=1 SV=4	191.383	63	29	53	29	531	cell organi	non-structu	nucleic aci	726779818
High	P02545	Prelamin-A/C OS=Homo sapiens OX=9606 GN=LMNA PE=1 SV=1	179.879	56	36	53	35	664	cell cycle (	cytosol;cy	cytoskeleta	792568860
High	Q15417	Calponin-3 OS=Homo sapiens OX=9606 GN=CNN3 PE=1 SV=1	178.878	60	16	85	14	329	cell organi	cytosol;cy	cytoskeleta	2964544631
High	Q8WX93	Palladin OS=Homo sapiens OX=9606 GN=PALLD PE=1 SV=3	177.516	31	33	48	33	1383	cell adhesi	plasma me	cytoskeleta	738178205
High	P29401	Transketolase OS=Homo sapiens OX=9606 GN=TKT PE=1 SV=3	161.438	47	27	43	27	623	other meta	other mem	other mole	633271782
High	P06748	Nucleophosmin OS=Homo sapiens OX=9606 GN=NPM1 PE=1 SV=2	155.886	39	11	41	11	294	cell cycle (	cytosol;cy	enzyme re	2539798252
High	P61978	Heterogeneous nuclear ribonucleoprotein K OS=Homo sapiens OX=9606 GN=HNRNPK PE=1 SV=1	154.238	44	18	66	18	463	RNA meta	cytoskeleto	nucleic aci	1877975233
High	Q05639	Elongation factor 1-alpha 2 OS=Homo sapiens OX=9606 GN=EEF1A2 PE=1 SV=1	150.27	44	17	96	4	463	protein me	other mem	nucleic aci	360099612.9
High	Q9Y450	HBS1-like protein OS=Homo sapiens OX=9606 GN=HBS1L PE=1 SV=1	139.637	50	26	36	26	684	cell organi	cytosol;tra	nucleic aci	297196413
High	P15311	Ezrin OS=Homo sapiens OX=9606 GN=EZR PE=1 SV=4	139.575	48	32	66	32	586	cell adhesi	plasma me	cytoskeleta	1273894929
High	Q08945	FACT complex subunit SSRP1 OS=Homo sapiens OX=9606 GN=SSRP1 PE=1 SV=1	139.288	39	22	43	22	709	cell organi	nucleus	nucleic aci	740463986
High	P05787	Keratin, type II cytoskeletal 8 OS=Homo sapiens OX=9606 GN=KRT8 PE=1 SV=7	137.844	60	32	57	25	483	cell organi	plasma me	other mole	666200698
High	Q14247	Src substrate cortactin OS=Homo sapiens OX=9606 GN=CTTN PE=1 SV=2	136.688	39	25	50	25	550	cell adhesi	plasma me	other mole	1572022288
High	Q86XP3	ATP-dependent RNA helicase DDX42 OS=Homo sapiens OX=9606 GN=DDX42 PE=1 SV=1	135.741	38	27	36	27	938	cell organi	cytosol;nu	nucleic aci	303427832
High	Q9Y2W1	Thyroid hormone receptor-associated protein 3 OS=Homo sapiens OX=9606 GN=THRAP3 PE=1 SV=2	127.264	25	23	38	22	955	RNA meta	nucleus	nucleic aci	423692024
High	P35269	General transcription factor IIF subunit 1 OS=Homo sapiens OX=9606 GN=GTF2F1 PE=1 SV=2	126.119	41	20	36	20	517	RNA meta	nucleus;ot	enzyme re	388612085
High	P06733	Alpha-enolase OS=Homo sapiens OX=9606 GN=ENO1 PE=1 SV=2	124.3	55	20	41	20	434	other meta	plasma me	nucleic aci	539707896
High	Q9Y4X5	E3 ubiquitin-protein ligase ARIH1 OS=Homo sapiens OX=9606 GN=ARIH1 PE=1 SV=2	121.625	36	18	30	18	557	protein me	cytosol;nu	other mole	325086831
High	P63244	Small ribosomal subunit protein RACK1 OS=Homo sapiens OX=9606 GN=RACK1 PE=1 SV=3	119.035	75	17	25	17	317	cell cycle (	plasma me	signal tran	347634207
High	O15371	Eukaryotic translation initiation factor 3 subunit D OS=Homo sapiens OX=9606 GN=EIF3D PE=1 SV=1	116.775	47	17	30	17	548	cell organi	cytosol;tra	nucleic aci	324046253
High	P60842	Eukaryotic initiation factor 4A-I OS=Homo sapiens OX=9606 GN=EIF4A1 PE=1 SV=1	114.677	58	22	39	20	406	protein me	cytosol;tra	nucleic aci	441200283
High	P23588	Eukaryotic translation initiation factor 4B OS=Homo sapiens OX=9606 GN=EIF4B PE=1 SV=2	113.187	38	24	54	24	611	protein me	cytosol;tra	nucleic aci	1457444917
High	P04406	Glyceraldehyde-3-phosphate dehydrogenase OS=Homo sapiens OX=9606 GN=GAPDH PE=1 SV=3	107.727	46	13	34	13	335	cell organi	plasma me	enzyme re	718986362
High	P13647	Keratin, type II cytoskeletal 5 OS=Homo sapiens OX=9606 GN=KRT5 PE=1 SV=3	105.589	45	30	54	15	590	cell organi	cytosol;cy	bone, tootl	339783592
High	Q07157	Tight junction protein ZO-1 OS=Homo sapiens OX=9606 GN=TJP1 PE=1 SV=3	103.984	16	24	32	24	1748	cell adhesi	plasma me	other mole	155315115
High	P02538	Keratin, type II cytoskeletal 6A OS=Homo sapiens OX=9606 GN=KRT6A PE=1 SV=3	100.006	44	27	56	1	564	cell organi	cytosol;cy	bone, tootl	79344694.3
High	P00338	L-lactate dehydrogenase A chain OS=Homo sapiens OX=9606 GN=LDHA PE=1 SV=2	99.895	48	16	33	15	332	other meta	cytosol;mi	other mole	465240273
High	P04259	Keratin, type II cytoskeletal 6B OS=Homo sapiens OX=9606 GN=KRT6B PE=1 SV=5	99.021	43	25	54	0	564	cell organi	cytosol;cy	bone, tootl	
High	P52597	Heterogeneous nuclear ribonucleoprotein F OS=Homo sapiens OX=9606 GN=HNRNPF PE=1 SV=3	98.557	41	12	36	10	415	RNA meta	plasma me	nucleic aci	625059444
High	Q15691	Microtubule-associated protein RP/EB family member 1 OS=Homo sapiens OX=9606 GN=MAPRE1 PE=	97.551	59	14	28	14	268	cell cycle (	plasma me	cytoskeleta	492788005
High	P02533	Keratin, type I cytoskeletal 14 OS=Homo sapiens OX=9606 GN=KRT14 PE=1 SV=4	96.659	54	22	38	7	472	cell organi	plasma me	cytoskeleta	406322365
High	Q9UHR5	SAP30-binding protein OS=Homo sapiens OX=9606 GN=SAP30BP PE=1 SV=1	95.766	55	16	30	16	308	other biolo	cytoskeleto		412341574
High	O60841	Eukaryotic translation initiation factor 5B OS=Homo sapiens OX=9606 GN=EIF5B PE=1 SV=4	95.47	20	19	29	19	1220	cell organi	cytosol;otl	nucleic aci	152098412
High	P06744	Glucose-6-phosphate isomerase OS=Homo sapiens OX=9606 GN=GPI PE=1 SV=4	94.316	36	17	29	17	558	other meta	non-structu	signal tran	593606662
High	P11142	Heat shock cognate 71 kDa protein OS=Homo sapiens OX=9606 GN=HSPA8 PE=1 SV=1	92.841	35	20	29	16	646	cell organi	non-structu	signal tran	157608036
High	Q13428	Treacle protein OS=Homo sapiens OX=9606 GN=TCOF1 PE=1 SV=3	92.754	18	22	29	22	1488	RNA meta	cytosol;nu	other mole	298862655
High	Q96HC4	PDZ and LIM domain protein 5 OS=Homo sapiens OX=9606 GN=PDLIM5 PE=1 SV=5	91.945	37	18	29	18	596	cell organi	other mem	cytoskeleta	322820115
High	Q06830	Peroxiredoxin-1 OS=Homo sapiens OX=9606 GN=PRDX1 PE=1 SV=1	90.008	75	15	33	14	199	cell cycle (	cytosol;nu	other mole	1434007117
High	O00267	Transcription elongation factor SPT5 OS=Homo sapiens OX=9606 GN=SUPT5H PE=1 SV=1	89.845	28	24	29	24	1087	cell organi	nucleus	nucleic aci	175880801
High	P39687	Acidic leucine-rich nuclear phosphoprotein 32 family member A OS=Homo sapiens OX=9606 GN=ANP32	88.392	36	13	47	7	249	transport;s	ER/Golgi;	other mole	2548672617
High	Q68CQ4	U3 small nucleolar RNA-associated protein 25 homolog OS=Homo sapiens OX=9606 GN=UTP25 PE=1 S	86.84	30	20	26	20	756	protein me	nucleus;ot	nucleic aci	171916957
High	Q96KG9	N-terminal kinase-like protein OS=Homo sapiens OX=9606 GN=SCYL1 PE=1 SV=1	85.38	29	19	22	19	808	stress resp	other mem	nucleic aci	194941377
High	P13984	General transcription factor IIF subunit 2 OS=Homo sapiens OX=9606 GN=GTF2F2 PE=1 SV=2	84.662	72	14	18	14	249	RNA meta	cytoskeleto	nucleic aci	346716489
High	Q9UJM3	ERBB receptor feedback inhibitor 1 OS=Homo sapiens OX=9606 GN=ERRFI1 PE=1 SV=1	84.288	50	15	30	15	462	cell cycle (	plasma me	enzyme re	240452491
High	P50454	Serpin H1 OS=Homo sapiens OX=9606 GN=SERPINH1 PE=1 SV=2	81.91	44	13	23	13	418	cell organi	non-structu	enzyme re	204484572
High	P26583	High mobility group protein B2 OS=Homo sapiens OX=9606 GN=HMGB2 PE=1 SV=2	79.806	44	9	27	7	209	cell organi	non-structu	signal tran	289925517
High	P23396	Small ribosomal subunit protein uS3 OS=Homo sapiens OX=9606 GN=RPS3 PE=1 SV=2	79.305	71	17	33	17	243	cell cycle (	plasma me	enzyme re	543712072
High	O60716	Catenin delta-1 OS=Homo sapiens OX=9606 GN=CTNND1 PE=1 SV=1	79.059	25	18	23	18	968	cell adhesi	plasma me	signal tran	103292612
High	Q14978	Nucleolar and coiled-body phosphoprotein 1 OS=Homo sapiens OX=9606 GN=NOLC1 PE=1 SV=2	78.88	25	17	40	17	699	cell cycle (	nucleus;ot	other mole	1151006472
High	Q92688	Acidic leucine-rich nuclear phosphoprotein 32 family member B OS=Homo sapiens OX=9606 GN=ANP32	77.6	34	12	42	6	251	cell organi	nucleus;ot	other mole	1773656449
High	Q9NYF8	Bcl-2-associated transcription factor 1 OS=Homo sapiens OX=9606 GN=BCLAF1 PE=1 SV=2	76.038	22	17	26	16	920	stress resp	nucleus;ot	nucleic aci	183377936
High	P08727	Keratin, type I cytoskeletal 19 OS=Homo sapiens OX=9606 GN=KRT19 PE=1 SV=4	75.677	52	20	27	13	400	cell organi	plasma me	cytoskeleta	262359097
High	Q99613	Eukaryotic translation initiation factor 3 subunit C OS=Homo sapiens OX=9606 GN=EIF3C PE=1 SV=1	75.442	22	20	25	20	913	cell organi	cytosol;tra	translation	175940023
High	P38919	Eukaryotic initiation factor 4A-III OS=Homo sapiens OX=9606 GN=EIF4A3 PE=1 SV=4	75.225	44	16	25	14	411	RNA meta	cytosol;nu	nucleic aci	169656420
High	Q8IXT5	RNA-binding protein 12B OS=Homo sapiens OX=9606 GN=RBM12B PE=1 SV=2	74.021	30	25	32	25	1001	other biolo	nucleus;ot		200639858
High	P62424	Large ribosomal subunit protein eL8 OS=Homo sapiens OX=9606 GN=RPL7A PE=1 SV=2	73.314	45	16	23	16	266	protein me	other mem	other mole	289027354
High	O00571	ATP-dependent RNA helicase DDX3X OS=Homo sapiens OX=9606 GN=DDX3X PE=1 SV=3	72.921	31	19	24	18	662	cell-cell si	non-structu	enzyme re	151943115
High	P08779	Keratin, type I cytoskeletal 16 OS=Homo sapiens OX=9606 GN=KRT16 PE=1 SV=4	72.882	39	17	27	6	473	cell organi	plasma me	cytoskeleta	27216555
High	Q9NW82	WD repeat-containing protein 70 OS=Homo sapiens OX=9606 GN=WDR70 PE=1 SV=1	71.317	27	16	22	16	654		nucleus;ot	other mole	113885017
High	Q15185	Prostaglandin E synthase 3 OS=Homo sapiens OX=9606 GN=PTGES3 PE=1 SV=1	70.666	53	9	45	9	160	cell cycle (	cytosol;nu	other mole	4580767025
High	P11586	C-1-tetrahydrofolate synthase, cytoplasmic OS=Homo sapiens OX=9606 GN=MTHFD1 PE=1 SV=4	70.645	30	23	26	22	935	other meta	cytosol;mi	other mole	187552820
High	P62805	Histone H4 OS=Homo sapiens OX=9606 GN=H4C16 PE=1 SV=2	68.784	53	8	39	8	103	cell organi	non-structu	nucleic aci	2462667422
High	P49411	Elongation factor Tu, mitochondrial OS=Homo sapiens OX=9606 GN=TUFM PE=1 SV=3	68.721	34	13	22	13	455	protein me	other mem	nucleic aci	231106703
High	Q99439	Calponin-2 OS=Homo sapiens OX=9606 GN=CNN2 PE=1 SV=4	67.553	43	11	27	9	309	cell organi	non-structu	cytoskeleta	443634771
High	P04083	Annexin A1 OS=Homo sapiens OX=9606 GN=ANXA1 PE=1 SV=2	65.229	43	12	15	12	346	cell-cell si	non-structu	signal tran	198765117
High	P46777	Large ribosomal subunit protein uL18 OS=Homo sapiens OX=9606 GN=RPL5 PE=1 SV=3	64.289	43	12	20	12	297	cell organi	cytosol;ER	enzyme re	186433170
High	P62495	Eukaryotic peptide chain release factor subunit 1 OS=Homo sapiens OX=9606 GN=ETF1 PE=1 SV=3	64.063	37	14	17	14	437	cell organi	cytosol;tra	translation	205723306
High	O95218	Zinc finger Ran-binding domain-containing protein 2 OS=Homo sapiens OX=9606 GN=ZRANB2 PE=1 SV	63.605	37	14	22	14	330	RNA meta	nucleus	other mole	379056493
High	Q14152	Eukaryotic translation initiation factor 3 subunit A OS=Homo sapiens OX=9606 GN=EIF3A PE=1 SV=1	61.971	16	19	25	19	1382	cell organi	cytosol;tra	nucleic aci	85579462.9
High	P17480	Nucleolar transcription factor 1 OS=Homo sapiens OX=9606 GN=UBTF PE=1 SV=1	60.502	18	12	18	12	764	cell organi	nucleus	nucleic aci	80518432
High	P09651	Heterogeneous nuclear ribonucleoprotein A1 OS=Homo sapiens OX=9606 GN=HNRNPA1 PE=1 SV=5	60.403	35	12	18	10	372	cell organi	cytosol;nu	nucleic aci	122833351
High	P00505	Aspartate aminotransferase, mitochondrial OS=Homo sapiens OX=9606 GN=GOT2 PE=1 SV=3	60.204	27	11	19	11	430	other meta	plasma me	other mole	110375861
High	P62906	Large ribosomal subunit protein uL1 OS=Homo sapiens OX=9606 GN=RPL10A PE=1 SV=2	60.177	40	9	18	9	217	protein me	cytosol;tra	other mole	242955987
High	P63241	Eukaryotic translation initiation factor 5A-1 OS=Homo sapiens OX=9606 GN=EIF5A PE=1 SV=2	59.256	53	8	15	8	154	protein me	other mem	translation	181629207
High	P27635	Large ribosomal subunit protein uL16 OS=Homo sapiens OX=9606 GN=RPL10 PE=1 SV=5	58.776	47	10	18	10	214	cell organi	cytosol;ER	other mole	148791048
High	P62701	Small ribosomal subunit protein eS4, X isoform OS=Homo sapiens OX=9606 GN=RPS4X PE=1 SV=2	58.295	54	15	19	15	263	protein me	cytosol;tra	nucleic aci	391121745
High	O75607	Nucleoplasmin-3 OS=Homo sapiens OX=9606 GN=NPM3 PE=1 SV=3	58.269	56	6	13	6	178	cell organi	cytosol;cy	other mole	234350366
High	P15880	Small ribosomal subunit protein uS5 OS=Homo sapiens OX=9606 GN=RPS2 PE=1 SV=2	56.646	51	14	21	14	293	protein me	cytosol;tra	nucleic aci	303122817
High	P50402	Emerin OS=Homo sapiens OX=9606 GN=EMD PE=1 SV=1	56.589	46	10	16	10	254	cell organi	other mem	cytoskeleta	119814605
High	P60228	Eukaryotic translation initiation factor 3 subunit E OS=Homo sapiens OX=9606 GN=EIF3E PE=1 SV=1	56.089	34	16	17	16	445	cell organi	cytosol;tra	nucleic aci	136433968
High	P61247	Small ribosomal subunit protein eS1 OS=Homo sapiens OX=9606 GN=RPS3A PE=1 SV=2	55.355	40	11	20	11	264	protein me	cytosol;ER	nucleic aci	303961970
High	P62995	Transformer-2 protein homolog beta OS=Homo sapiens OX=9606 GN=TRA2B PE=1 SV=1	55.117	31	8	19	7	288	RNA meta	other mem	nucleic aci	307032968
High	P62136	Serine/threonine-protein phosphatase PP1-alpha catalytic subunit OS=Homo sapiens OX=9606 GN=PPP1C	54.893	34	9	11	2	330	cell adhesi	plasma me	other mole	44239538.4
High	P04183	Thymidine kinase, cytosolic OS=Homo sapiens OX=9606 GN=TK1 PE=1 SV=2	54.708	53	10	16	10	234	cell cycle (	cytosol;nu	kinase acti	280993976
High	Q00839	Heterogeneous nuclear ribonucleoprotein U OS=Homo sapiens OX=9606 GN=HNRNPU PE=1 SV=6	54.24	16	12	20	12	825	cell cycle (	cytosol;cy	cytoskeleta	244857258
High	P20700	Lamin-B1 OS=Homo sapiens OX=9606 GN=LMNB1 PE=1 SV=2	52.477	27	14	17	10	586	cell organi	other mem	cytoskeleta	75223879.6
High	Q9H4B7	Tubulin beta-1 chain OS=Homo sapiens OX=9606 GN=TUBB1 PE=1 SV=1	52.438	11	7	92	1	451	cell adhesi	cytoskeleto	cytoskeleta	4812103.5
